# Deconstruction of a hypothalamic astrocyte-white adipocyte sympathetic axis that regulates lipolysis in mice

**DOI:** 10.1038/s41467-022-35258-6

**Published:** 2022-12-07

**Authors:** Dan Chen, Yong Qi, Jia Zhang, Yunlei Yang

**Affiliations:** 1 Department of Medicine Division of Endocrinology, Albert Einstein College of Medicine, Bronx, NY 10461 USA; 2 Department of Respiratory and Critical Care Medicine, Henan Provincial People’s Hospital, People’s Hospital of Zhengzhou University, Zhengzhou, Henan 450003 China; 3 Department of Neuroscience, Albert Einstein College of Medicine, Bronx, NY 10461 USA; 4 Einstein-Mount Sinai Diabetes Research Center, Albert Einstein College of Medicine, Bronx, NY 10461 USA; 5 The Fleischer Institute for Diabetes and Metabolism, Albert Einstein College of Medicine, Bronx, NY 10461 USA

**Keywords:** Neuroscience, Endocrinology, Metabolism

## Abstract

The role of non-neuronal glial cells in the regulation of adipose sympathetic nerve activity and adipocyte functions such as white adipose tissue lipid lipolysis is poorly understood. Here, we combine chemo/optogenetic manipulations of medio-basal hypothalamic astrocytes, real-time fiber photometry monitoring of white adipose tissue norepinephrine (NE) contents and nerve activities, electrophysiological recordings of local sympathetic inputs to inguinal white adipose tissue (iWAT), and adipose tissue lipid lipolytic assays to define the functional roles of hypothalamic astrocytes in the regulation of iWAT sympathetic outflow and lipolysis. Our results show that astrocyte stimulation elevates iWAT NE contents, excites sympathetic neural inputs and promotes lipolysis. Mechanistically, we find that sympathetic paravertebral ganglia (PG) partake in those astrocyte effects. We also find that astrocyte stimulation excites *pro-opiomelanocortin* (POMC) neurons in the arcuate nucleus of the hypothalamus (ARH), and chemogenetic inhibition of POMC neurons blunts the effects induced by astrocyte stimulation. While we cannot exclude potential roles played by other cell populations such as microglia, our findings in this study reveal a central astrocyte-peripheral adipocyte axis modulating sympathetic drive to adipose tissues and adipocyte functions, one that might serve as a target for therapeutic intervention in the treatment of obesity.

## Abstract (web summary)

The role of non-neuronal glial cells in the regulation of adipose tissue function are incompletely understood. Here the authors report that stimulation of mediobasal hypothalamic astrocytes promotes inguinal white adipose tissue lipolysis in mice, dependent on the activation of the pro-opiomelanocortin neurons.

## Introduction

Precise control of adipose tissue lipid metabolism is both necessary and important for the regulation of energy homeostasis and body weight, as its dysfunctions may lead to metabolic disorders such as obesity and diabetes^1–3^. Obesity comprises an excessive amount of body fat mass. However, effective treatments for obesity and its comorbidities remain lacking, which might be attributable to our incomplete understanding of the implicated central and peripheral processes that orchestrate sympathetic drive to adipose tissues and adipocyte functions. There is ample evidence to indicate that the sympathetic nervous system (SNS) exerts important and significant roles in modulating adipocyte functions including white adipose tissue lipid lipolysis^4–12^. To combat obesity and its comorbidities, it thus is of significance in deciphering and manipulating previously unknown brain–adipose signaling pathways that promote adipose sympathetic strength. Previous studies have been focused on neuronal mechanisms underlying adipose tissue lipid metabolism^13^; however, an essential but poorly understood role is that played by glial cells in the central nervous system (CNS), particularly in the hypothalamus.

Hence, we set off to study whether and how hypothalamic astrocytes modulate sympathetic drives to inguinal white adipose tissue (iWAT) and to identify the involved central sympathetic outflow neurons and peripheral sympathetic ganglia. To selectively manipulate hypothalamic astrocytes and evaluate peripheral adipose tissue sympathetic strength, we utilized a viral vector-assisted approach, fiber photometry monitoring of adipose tissue sympathetic norepinephrine (NE) contents and nerve firing rates, and electrophysiological recordings of sympathetic nerves in live mice and ex vivo tissues respectively. For example, we took advantage of coordinated combined real-time fiber photometry monitoring of iWAT NE contents using the GPCR activation-based NE (GRAB_NE) sensor^14^ and Ca^2+^-dependent GCaMP signals, circumventing the long-standing limitation to detect and evaluate dynamics of sympathetic inputs to adipose tissues in live animals. These cutting-edging methods give us both neuroanatomically and temporally specific access to defining the roles played by hypothalamic astrocytes in scaling adipose tissue sympathetic strength and adipocyte functions.

Here, we show that acute stimulation of the glial fibrillary acidic protein (GFAP)-expressing hypothalamic glial cells (hereafter referred to as astrocytes) in the ARH promotes the sympathetic drive to iWAT and increases lipolysis. We also find that POMC neurons in the ARH and paravertebral sympathetic ganglia (PG) are involved in the astrocyte stimulation-induced effects. Collectively, we demonstrate a previously unprecedented central astrocyte–peripheral adipocyte connection that modulates iWAT, filling in an important gap in our understanding of glial regulations of adipocytes.

## Results

### Validation of astrocyte selectivity of viral transductions and astrocyte stimulation

**Fig. 1:**
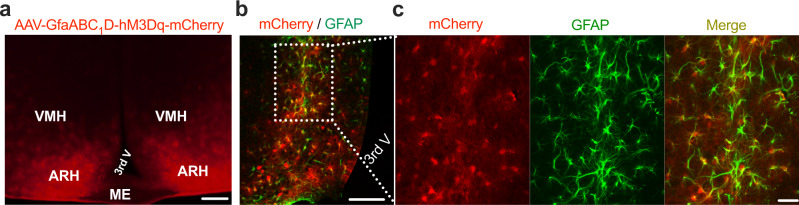
Astrocyte selective expressions of DREADDs. **a** A representative confocal image of viral transductions of GfaABC_1_D-dependent hM3Dq-mCherry in the ARH. Almost all the mCherry-transduced cells (87 ± 16.6/five sections under ×40 objective, *n* = 6) are GFAP-positive. **b**, **c** Representative images showing colocalizations of mCherry-transduced cells (red) and Alexa fluor 488 anti-GFAP antibody-labeled cells (green). The dotted line in **b** indicates the region shown in (**c**). Scale bars for **a** 1 mm, **b** 200 μm, **c** 25 μm. ARH, arcuate nucleus of the hypothalamus; ME median eminence, VMH ventromedial hypothalamus, 3rd V third ventricle.

We validated the chemogenetic Designer Receptor Exclusively Activated by Designer Drug (DERADD)-based astrocyte stimulation with real-time photometry monitoring astrocytic Ca^2+^ signals in live mice. We virally transduced ARH astrocytes following previously documented procedures^15–17^. Using a tiny capillary injector, we targeted a vector carrying GfaABC_1_D-dependent hM3Dq to the ARH (Fig. 1a). Consistent with the previous studies^18–20^, we verified the astrocyte selectivity of the GfaABC_1_D-dependent vectors, as evidenced by the predominantly virally transduced GFAP-positive astrocytes (Fig. 1b, c) with no or little transductions in neurons (Supplementary Fig. 1) or microglia (Supplementary Fig. 2). To evaluate DREADD-based astrocyte stimulation, we targeted two viral vectors, respectively, carrying GfaABC_1_D-dependent hM3Dq and GCaMP_6f_^18,19^ to the ARH and implanted a photometry fiber over the ARH (Supplementary Fig. 3a–c). Two weeks after the transductions and one week of acclimating to both intraperitoneal injection (i.p.) procedures and carrying the optic cable connected to the implanted fiber, we performed real-time photometry monitoring of astrocytic GCaMP_6f_ signals. A single i.p. injection of a selective DREADD agonist J60 elevated Ca^2+^ levels in the ARH astrocyte hM3Dq-transduced mice compared to control mice (Supplementary Fig. 3d–g).

### Verification of real-time photometry monitoring of dynamics of iWAT sympathetic outflows

It is a longstanding limitation to performing real-time measurements of peripheral tissue sympathetic outflows in live animals. To overcome this limitation, we took advantage of recently developed and established fiber photometry and GRAB_NE sensor. The GRAB_NE sensor is a genetically encoded GPCR-activated GFP indicator with rapid detection and high specificity for NE^14^. Although it also is possible to detect NE derivative epinephrine (Epi), it is noteworthy to point out that the enzyme phenylethanolamine N-methyltransferase (PNMT) converting NE to Epi is primarily located in the adrenal gland of mammals with no detectable PNMT mRNA in normal adipose tissues^21^. We thus expect that the increased intensity of GRAB_NE signals is primarily mediated by the increased amount of NE in adipose tissues.

**Fig. 2:**
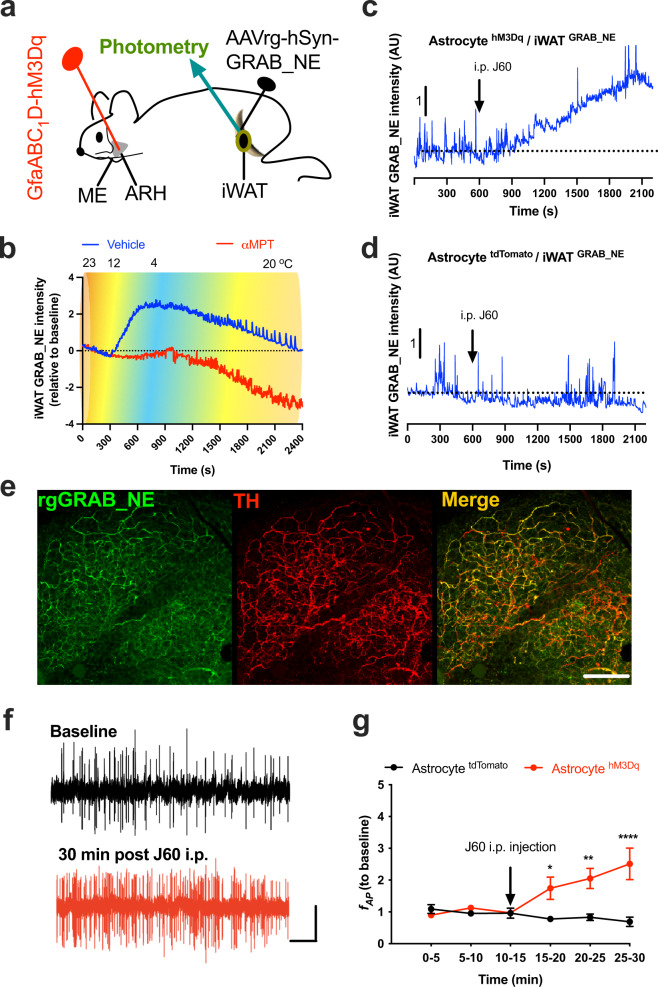
Astrocyte stimulation increases iWAT sympathetic drive. **a** Schematic illustration of targeting a vector carrying GfaABC_1_D-dependent hM3Dq to the ARH and a second retrograde vector carrying hSyn-dependent GRAB_NE to the iWAT, and of implanting a photometry cannula in the injected iWAT. **b** Representative traces of real-time wireless photometry monitoring of iWAT NE contents with vehicle (blue) or αMPT (red) treatment. **c**, **d** Astrocyte stimulation with J60 via i.p. injection increased the intensity of iWAT GRAB_NE signals in the astrocyte **c** hM3Dq- but not in **d** tdTomato-transduced mice. **e** Representative confocal images of virally transduced iWAT GRAB_NE (green) and Alexa Fluo 647-conjugated anti-TH antibody labeled nerves (red). **f**, **g** Astrocyte stimulation increases the firing rates of local sympathetic inputs to iWAT: **f** Representative traces of nerve spikes at baseline and 30 min post J60 treatment via i.p in astrocyte hM3Dq-transduced mice; **d** Group data of iWAT nerve firing rates recorded in astrocyte hM3Dq or tdTomato-transduced mice (*n* = 6 per group). TH tyrosine hydroxylase. Two-way ANOVA with Sidak post hoc tests; data represent mean ± s.e.m.; *p = 0.0187 < 0.05; **p = 0.0015 < 0.01; ****p < 0.0001. Scale bars for **e** 100 μm, and **f** 10 s and 30 μV. Arrows in **c**, **d**, **g** indicate the J60 i.p. injection time point. ARH arcuate nucleus of the hypothalamus, ME median eminence, iWAT inguinal white adipose tissue.

Following previously published procedures^22^, we made an incision along the dorsal–ventral axis of the iWAT incision to expose the dorsolumbar portion of the iWAT relative to the inguinal portion of the iWAT. The prominent lymph node in the iWAT was used as an anatomical marker to separate the dorsolumbar from the inguinal iWAT portions. We injected a retrograde vector carrying the hSyn-dependent GRAB_NE sensor across the dorsolumbar iWAT. Two weeks after the injections and 3 days prior to performing the tests, to minimize locomotor activity, we cut the femoral nerve passing under iWAT and innervating anterior thigh muscles, and implanted a photometry cannula in the previously injected dorsolumbar iWAT (Fig. 2a). We validated our photometry approach by placing the virally transduced and implanted mice in a temperature-controlled chamber. There is evidence to indicate that cold challenge increases iWAT sympathetic drive^23^. Consistently, our real-time photometry results show that cold potently increased the intensity of iWAT GRAB_NE signals (Fig. 2b). To further validate our photometry approach and the GRAB_NE sensor, we treated mice with a tyrosine hydroxylase (TH) inhibitor αMPT to inhibit NE production. Following previously documented mouse protocols^24^, we treated the mice with a first i.p. injection of αMPT (100 mg/kg) at 4 h and a second i.p. injection (50 mg/kg) at 2 h before placing the mice in the cold chamber. We observed that prior inhibition of NE production with the TH inhibitor blunted the cold-induced effects (Fig. 2b and Supplementary Fig. 4a). Together, these results demonstrate that our combined photometry and GRAB_NE approach can be reliably used to evaluate the dynamics of adipose sympathetic NE contents in a spatiotemporal manner.

### Chemogenetic stimulation of ARH astrocytes elevates iWAT NE contents

We next examined the ability of astrocytes to modulate sympathetic drive to iWAT. We targeted a vector carrying the GfaABC_1_D-dependent hM3Dq to the ARH and a second retrograde vector carrying hSyn-dependent GRAB_NE to inguinal iWAT. It is technically challenging in implanting a photometry fiber in inguinal iWAT or manipulating those paravertebral sympathetic ganglia (PG) above the diagram in live animals, so we next focused to evaluate iWAT sympathetic drive in lightly sedated mice under a light plane of anesthesia. Two weeks after the viral transfections, mice were anesthetized and a photometry fiber was placed on the iWAT entry point of the anterior cutaneous femoral nerve (ACFN) branch under a stereomicroscope. Mice were then maintained in a lightly sedated condition. We observed that astrocyte stimulation with a single i.p. injection of J60 increased the intensity of GRAB_NE signals in the astrocyte hM3Dq-transduced mice (Fig. 2c and Supplementary Fig. 3b) compared to tdTomato-transduced mice (Fig. 2d and Supplementary Fig. 4c). We next performed post-hoc evaluations of GRAB_NE expressions in iWAT. Following published methods^6,13,25^, we harvested and cleared the virally injected iWAT and stained it with anti-TH antibodies (Fig. 2e).

### Astrocyte stimulation excites local sympathetic inputs to iWAT

We next examined whether astrocyte stimulation modulates iWAT-supporting local sympathetic inputs. We first virally transduced ARH astrocytes with the stimulatory hM3Dq. Following published protocols^26^ with some modifications, the virally transduced mice were anesthetized with isoflurane through a face mask and placed in a surgical platform. We isolated the iWAT-supporting ACFN branch and severed distal to the electrode placement site to disconnect afferent fibers, and the efferent (proximal) end of the nerve was placed on two coated silver hook electrodes. Mice were then maintained under a light plane of anesthesia. One hour after a stable light anesthetic state, we performed electrophysiological recordings of the nerve’s electrical activity. We observed that astrocyte stimulation with J60 via i.p. increased the firing rates of iWAT-supporting ACFN in the astrocyte hM3Dq-transduced mice compared to the tdTomato-transduced mice (Fig. 2f, g).

**Fig. 3:**
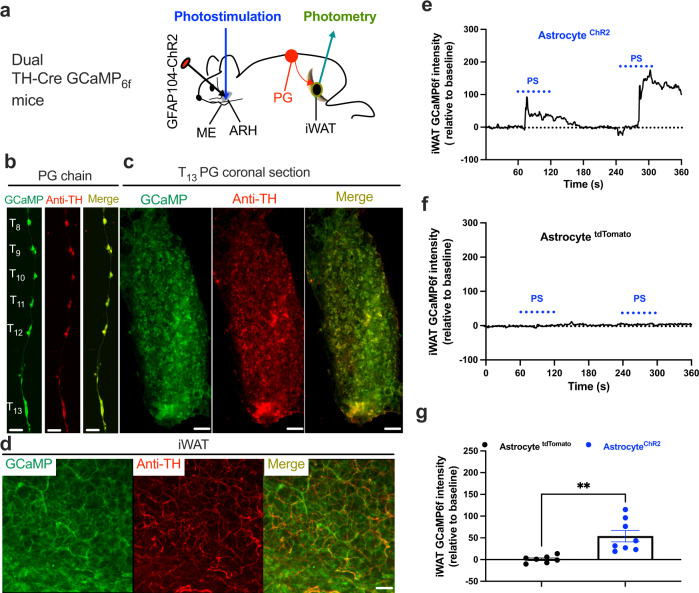
Photostimulation of ARH astrocytes excites sympathetic inputs to iWAT. **a** Schematic illustration of targeting a vector carrying GFAP104-dependent ChR2 to the ARH in dual TH-Cre GCaMP_6f_ mice and implanting an optic fiber over the ARH for photostimualtion (PS) of ChR2-transduced astrocytes in the ARH, and two weeks later placing a photometry fiber on the ACFN entry point into the inguinal iWAT for photometry monitoring of Ca^2+^-dependent GCaMP_6f_ signals in lightly sedated mice. **b**–**d** Representative images showing GCaMP_6f_ expressions and TH stain in **b** paravertebral sympathetic chain, **c** coronal sections of T_13_ PG, and **d** inguinal iWAT. **e**-**g** PS of astrocytes excited sympathetic neural inputs in the inguinal iWAT: Representative traces of photometry monitoring of iWAT GCaMP_6f_ signals with PS of astrocytes in the **e** ChR2- and **f** tdTomato-transduced dual TH-Cre GCaMP_6f_ mice; and **g** Group data of average GCaMP_6f_ signals (tdTomato, *n* = 7; ChR2, *n* = 8). Two-tailed Student’s *t*-tests; data represent mean ± s.e.m.; **p = 0.0025 (*t* = 3.727, df = 13)<0.01. Scale bars for **b** 2 mm and **c** 50 μm. PG paravertebral ganglia. Blue dotted lines in **e** and **f** indicated the PS duration (20 Hz for 1 s; repeated every 4 s for 60 s).

We next focused on astrocyte stimulation with complementary optogenetic stimulation in transgenic animals. We used optogenetics for photostimulation (PS) of blue light-sensitive ChR2-transduced astrocytes via an implanted optic cannula over the ARH, and evaluated the activity of iWAT-supporting sympathetic nerves in dual TH-Cre GCaMP_6f_ mice (Fig. 3a and Supplementary Fig. 5). We validated Cre-dependent GCaMP_6f_ expressions in TH-positive neurons in paravertebral ganglia and nerves in inguinal iWAT in dual TH-Cre GCaMP_6f_ mice. We transcardially perfused the mice and harvested the PG chain and inguinal iWAT, which were cryo-sectioned for imaging. We stained the sections with anti-TH antibodies. Dense expressions of GCaMP_6f_ were observed in about 70% of TH-positive neurons in the PG (Fig. 3b, c) and TH-positive nerves in iWAT (Fig. 3d).

In cohort groups of mice, we transduced ARH astrocytes with the stimulatory ChR2 and implanted an optic cannula over the ARH which was connected to a blue laser via an optic cable. Two weeks after viral transductions, we anesthetized the mice and placed a photometry fiber at the iWAT entry point of the ACFN branch for real-time photometry monitoring of adipose nerve GCaMP_6f_ signals in lightly sedated mice. Consistently, we observed that photostimulation of ChR2-transduced astrocytes increased the intensity of iWAT GCaMP_6f_ signals in the astrocyte ChR2-transduced mice (Fig. 3e, g) compared to tdTomato-transduced mice (Fig. 3f, g). These results suggest that astrocyte stimulation excites iWAT-supporting sympathetic outflow neurons.

### Photostimulation of PG excites iWAT-supporting postganglionic sympathetic neurons

**Fig. 4:**
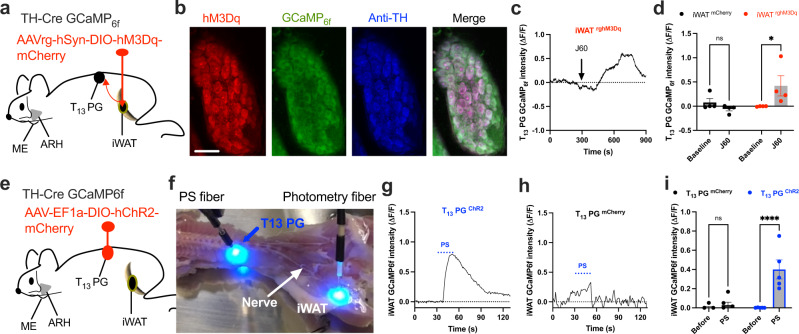
Functional evidence of T_13_ PG providing sympathetic inputs to inguinal iWAT. **a** Schematic illustration of targeting a retrograde vector carrying Cre-dependent hM3Dq-mCherry to inguinal iWAT in dual TH-Cre GCaMP_6f_ mice. **b** Sample images showing colocalizations of retrograde traced and hM3Dq-mCherry (red)-transduced TH neurons stained with Alex Fluor 350-conjugated anti-TH antibodies (blue) which also expressed GCaMP_6f_ (green) in T_13_ PG. **c**, **d** Ex vivo photometry monitoring was performed at acute isolated T_13_ PG from the retrograde traced and hM3Dq-transduced TH-Cre GCaMP_6f_ mice, and chemogenetic stimulation of the hM3Dq-transduced TH neurons with J60 addition to the circulating aCSF increased the intensity of GCaMP_6f_ signals in the T_13_ PG: **c** A representative trace of real-time monitoring of T_13_ PG GCaMP_6f_ signals with J60 addition as pointed; and **d** Group data of T_13_ PG GCaMP_6f_ signals in iWAT injected with hM3Dq- or mCherry (*n* = 4 per group). **e**–**i** PS of ChR2-transduced T_13_ PG excited the sympathetic neural inputs in ex vivo inguinal iWAT: **e** Illustration of targeting a vector carrying Cre-dependent ChR2 to T_13_ PG in dual TH-Cre GCaMP_6f_ mice; **f** Ex vivo intact spinal cord-iWAT tissues with a PS fiber placed on exposed T_13_ ganglion and a photometry fiber placed on the ACFN entry point in inguinal iWAT in a recording chamber; **g**–**i** Representative photometry monitoring of GCaMP_6f_ signals with PS of T_13_ PG **g** ChR2- and **h** mCherry-transduced mice, and **i** Group data of average GCaMP_6f_ signals from **g** and **h** (mCherry, *n* = 4; ChR2, *n* = 5). Two-way ANOVA with Sidak post hoc tests; data represent mean ± s.e.m; *p* = 0.67 (**d**, mCherry); *p = 0.04 < 0.05 (**d**, rghM3Dq); *p* = 0.91 (**i**, mCherry); ****p < 0.0001 (**i**, ChR2); n.s. (not significant). Scale bars for **b** 50 μm. PG, paravertebral ganglia. Arrow in **c** indicated the addition of J60 in the circulating aCSFs. Blue dotted lines in **g** and **h** indicated the PS duration (20 Hz for 1 s; repeated every 4 s for 30 s).

The neurobiological link between central astrocyte and peripheral adipocyte remained unclear and the involved sympathetic ganglia await identification. We performed experiments to retrograde trace and stimulate inguinal iWAT-projecting ganglia containing sympathetic postganglionic neurons. We targeted a retrograde vector carrying Cre-dependent hM3Dq to the inguinal iWAT in dual TH-Cre GCaMP_6f_ mice (Fig. 4a). Two weeks after viral transductions, we perfused the mice and extracted the PG at the level of the 13th thoracic vertebra (T_13_ PG). We sectioned and imaged the extracted PG. To verify hM3Dq-mCherry expressions on TH-positive neurons, we stained the sections with anti-TH antibodies and observed dense hM3Dq expressions on TH-positive neurons (Fig. 4b). We next developed and validated ex vivo real-time photometry monitoring of GCaMP_6f_ signals. We acutely extracted the T_13_ PG and transferred the ganglia to a recording chamber containing 34 °C oxygenated (95% O_2_−5% CO_2_) artificial cerebrospinal fluids (aCSFs). A photometry fiber was placed on the ganglia. After a stable baseline recording, the addition of J60 to the circulating aCSFs increased the intensity of ganglia GCaMP_6f_ signals in the iWAT rghM3Dq-transduced mice compared to controls (Fig. 4c, d). This result suggests that the T_13_ PG contains the inguinal iWAT-projecting postganglionic neurons.

Furthermore, we performed functional experiments to selectively stimulate ChR2-transduced TH neurons localized within the T_13_ PG and simultaneously monitored GCaMP_6f_ -expressing neural inputs in ex vivo inguinal iWAT. Following published protocols^27,28^, we made a ventro-dorsal incision over the left kidney and pushed the kidney down to expose the T_13_ PG in dual TH-Cre GCaMP_6f_ mice. We targeted a vector carrying Cre-dependent ChR2 to the T_13_ ganglion with a micromanipulator-controlled tiny capillary needle (Fig. 4e). We sectioned and stained the injected PG and iWAT to validate viral transfections in PG and iWAT. We observed that about half of the GCaMP_6f_-positive neurons expressed the mCherry in the injected PG (Supplementary Fig. 6a) and colocalizations of mCherry and GCaMP_6f_ in iWAT (Supplementary Fig. 6b). As independent experiments, we transcardially perfused cohort groups of virally transduced mice with cold (4 °C) oxygenated aCSFs and acutely dissected the whole intact spinal cord-iWAT ex vivo tissues, which were subsequently incubated in a chamber containing the aCSFs at 34 °C for 30 min before transferring to a submersion-recording chamber. We placed a photostimulation fiber on the exposed T_13_ ganglion and a photometry fiber on the iWAT entry point of the ACFN branch (Fig. 4f), respectively for photostimulation of ChR2-transduced neurons in the ganglion and photometry monitoring of iWAT nerve GCaMP_6f_ signals. Photostimulation of ChR2-transduced neurons in the T_13_ ganglion increased the intensity of GCaMP_6f_ signals in ChR2-transduced mice compared to mCherry-transduced control mice (Fig. 4g–i).

### T_13_-L_1_ PG partake in the astrocytic promotion of sympathetic outflows to iWAT

**Fig. 5:**
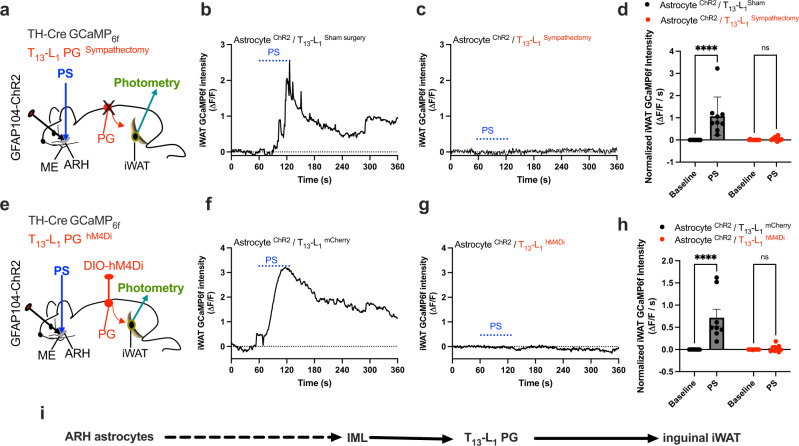
T_13_-L_1_ PG sympathectomy or inhibition blunts astrocyte promoting the sympathetic drive to iWAT. **a** Schematic illustration of targeting a vector carrying GFAP104-dependent ChR2 to the ARH and implanting an optic fiber over ARH, and placing a photometry fiber in inguinal iWAT in T_13_-L_1_ PG sympathectomy dual TH-Cre GCaMP_6f_ mice. **b**–**d** PG sympathectomy blunts astrocyte stimulation-induced excitation of iWAT sympathetic inputs: **b**, **c** Representative photometry monitoring of TH-positive nerve GCaMP_6f_ signals with PS of ChR2-transduced astrocytes in the PG **b** Sham- and **c** sympathectomy mice respectively, and **d** Group data of average GCaMP_6f_ signals from **b** and **c** (*n* = 9 per group). **e** Schematic illustration of targeting a vector carrying Cre-dependent hM4Di to T_13_-L_1_ PG in astrocyte ChR2-transduced and ARH-implanted dual TH-Cre GCaMP_6f_ mice. **f**-**h**: Inhibition of the PG with J60 via i.p. 30 min prior to performing photometry blunted the PS-induced effects: Representative photometry monitoring of TH-positive nerve GCaMP_6f_ signals with PS of ChR2-transduced astrocytes in the PG **f** mCherry - and **g** hM4Di-transduced mice, and **h** Group data of average GCaMP_6f_ signals from **f** and **g** (n = 8 per group). **i** A hypothetical neural pathway for central astrocyte regulation of iWAT through the PG. Two-way ANOVA with Sidak post hoc tests; data represent mean ± s.e.m.; ****p < 0.0001 (**d**, Astrocyte^ChR2^/T_13_-L_1_^Sham^); *p* = 0.99 (**d**, Astrocyte^ChR2^ /T_13_-L_1_^Sympathectomy^); ****p < 0.0001 (**h**, Astrocyte^ChR2^/T_13_-L_1_^mCherry^); *p* = 0.94 (**h**, Astrocyte^ChR2^/T_13_-L_1_^hM4Di^); n.s. (not significant). Blue dotted lines in **b, c, f**, and **g** indicated the PS duration (20 Hz for 1 s; repeated every 4 s for 60 s).

We next examined the involvements of the PG at T_13_ and lumbar vertebral level 1 (L_1_) in the astrocytic regulations of iWAT sympathetic outflows. A recent study shows that the L_1_ PG also innervates the inguinal iWAT^25^. We first transduced ARH astrocytes with the stimulatory ChR2 in dual TH-Cre GCaMP_6f_ mice, and implanted an optic fiber over the injected ARH (Fig. 5a). To achieve ganglia sympathectomy, following previously published procedures^27,28^ we decentralized both T_13_ and L_1_ PG and cut the relevant white ramus and gray ramus. The spinal nerves and blood vessels were left intact. Three days after the surgical PG sympathectomy, we placed a photometry fiber at the entry point of the ACFN branch in ipsilateral inguinal iWAT in lightly sedated mice. PS of ARH astrocytes increased the intensity of iWAT GCaMP_6f_ signals in the astrocyte ChR2-transduced and PG Sham-treated mice (Fig. 5b, d) but not in the PG sympathectomy mice (Fig. 5c, d). To verify the results obtained from the ganglia sympathectomy, we transduced T_13_-L_1_ PG with the inhibitory DREADD-hM4Di in cohort groups of the astrocyte ChR2 transduced and both ARH and iWAT implanted TH-Cre GCaMP_6f_ mice (Fig. 5e). J60 administration via i.p. 30 min prior to PS of ARH astrocytes blunted those astrocyte effects in the PG hM4Di-transduced mice compared to PG mCherry-transduced mice (Fig. 5f–h). These results provide functional evidence that both T_13_-L_1_ PG supports inguinal iWAT and partake in the astrocytic regulation of iWAT sympathetic drive (Fig. 5i).

### Astrocyte stimulation induces iWAT lipolysis

**Fig. 6:**
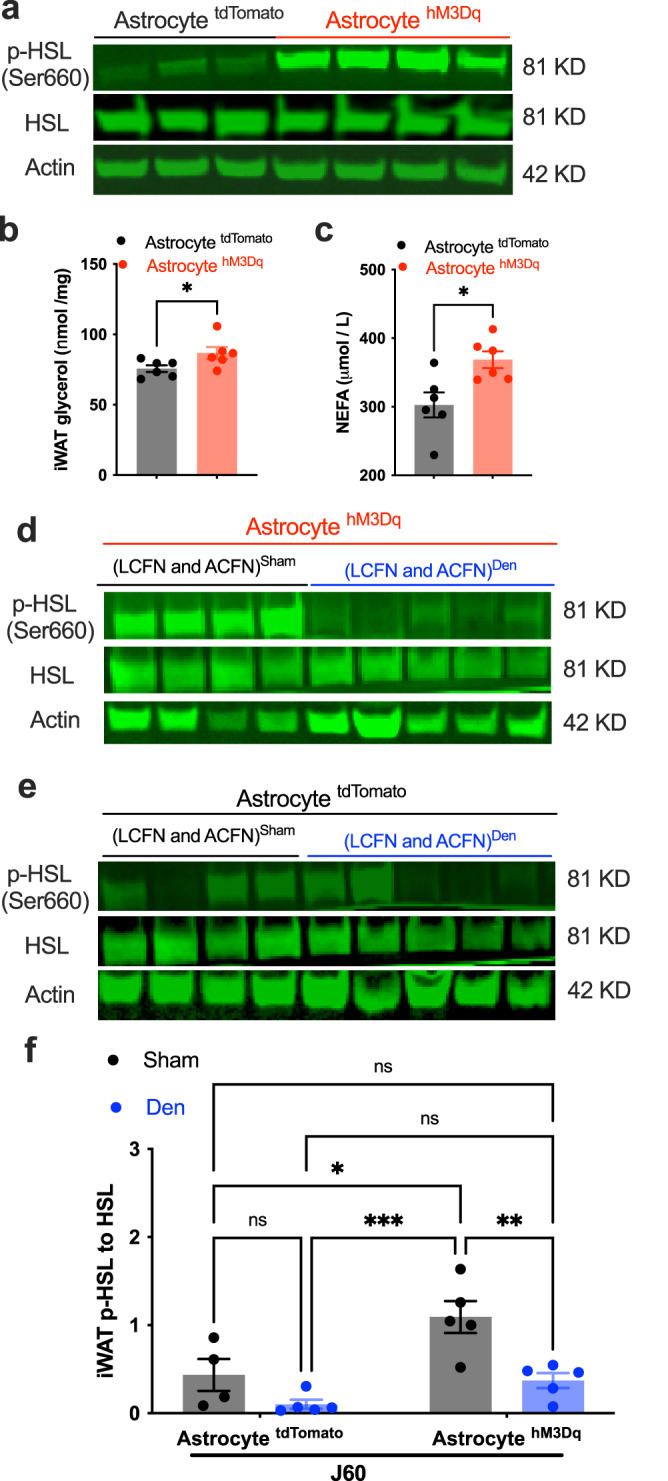
Local neural inputs are required for astrocyte regulation of lipolysis. **a**–**c** Chemogenetic stimulation of astrocytes in the ARH with J60 via i.p. induced iWAT lipolysis in the astrocyte hM3Dq-transduced mice compared to tdTomato-transduced controls, as evidenced by increased levels of p-HSL and glycerol and plasma NEFA: **a** Representative western blots of p-HSL and HSL and actin in inguinal iWAT extracted 2 h post a single i.p. injection of J60 in astrocyte tdTomato or hM3Dq-transduced mice; **b**, **c** Group data of **b** iWAT glycerol level and **c** plasma NEFA (*n* = 6 per group). **d–f** LCFN and ACFN physical denervation blunted the astrocyte-stimulation-induced HSL phosphorylation: **d**, **e** Representative blots of p-HSL and HSL and actin in inguinal iWAT extracted 2 h post a single i.p. injection of J60, from astrocyte **d** hM3Dq- or **e** tdTomato-transduced mice which received a control Sham or crushing injury to iWAT-supporting LCFN and ACFN; and **f** Group data of p-HSL over HSL from astrocyte tdTomato or hM3Dq-transduced mice with sham or crushing surgeries respectively (Tdtomato^Sham^, *n* = 4; Tdtomato^Den^, *n* = 5; hM3Dq^Sham^, *n* = 5; hM3Dq^Den^, *n* = 5). Data represent mean ± s.e.m; two-tailed Student’s *t*-tests for **b** (*p* = 0.04) and **c** (*p* = 0.01); two-way ANOVA with Sidak post hoc tests for **f** (tdTomato^Sham to Den^, *p* = 0.48; Sham^tdTomato to hM3Dq^, *p* = 0.02; tdTomato^Sham^ to hM3Dq^Den^, *p* = 0.99; tdTomato^Den^ to hM3Dq^Sham^, *p* = 0.0004; tdTomato^Sham^ to hM3Dq^Den^, *p* = 0.99; tdTomato^Den^ to hM3Dq^Den^, *p* = 0.65; hM3Dq^Sham to Den^, *p* = 0.008; *p < 0.05; **p < 0.01; ***p < 0.001; ns, not significant.

We examined the ability of ARH astrocytes to modulate lipolysis. We treated the astrocyte hM3Dq-transduced mice with J60 via a single i.p. injection in the morning when animals usually do not eat and removed the food from the cage two h before the injection. Two hours post the injection, we performed retro-orbital blood collections and extracted inguinal iWAT, which were used, respectively, for the detection of plasma-free fatty acids and measurements of adipose tissue glycerol and lipolytic enzyme. Astrocyte stimulation increased activation (S660) site phosphorylation of hormone-sensitive lipase (HSL) (Fig. 6a), adipose tissue levels of glycerol (Fig. 6b), and plasma levels of non-esterified fatty acids (NEFA) (Fig. 6c) in the astrocyte hM3Dq-transduced mice compared to tdTomato-transduced controls. To evaluate the potential for astrocytes to control the gene markers of lipolysis, we measured the mRNA levels of two key markers of lipolysis Hsl and adipose triglyceride lipase (Atgl). Astrocyte stimulation did not significantly affect gene expressions of the Hsl and Atgl (Supplementary Fig. 7a). We also assayed the gene expressions of adipocyte browning and thermogenic marker Ucp1 (Uncoupling protein 1) and additional thermogenic factors (Dio2; Prdm16). Astrocyte stimulation did not elicit effects on the mRNA expressions of these thermogenic markers (Supplementary Fig. 7b). Matching with the astrocyte effect on exciting iWAT-supporting sympathetic nerves, these results suggest that astrocyte stimulation induces a rapid neuron activity-dependent release of sympathetic NE into the iWAT, which subsequently increases protein kinase A (PKA) phosphorylation of HSL via beta-adrenergic stimulation, while this type of astrocyte stimulation and local sympathetic inputs are probably not sufficient to induce transcription and translation of genes encoding lipolytic enzymes.

### Local neural inputs are required for ARH astrocyte regulations of HSL phosphorylation

To disrupt the inguinal iWAT-innervating nerves, we performed a local neural crush injury or sham surgery both to the lateral cutaneous femoral nerve (LCFN) and to the ACFN branches that provide neural inputs to the inguinal iWAT^25^. We carried out physical denervation with forceps crushing the two branches about 2 mm from the distal iWAT entry points respectively. Three days post-surgery, we treated the iWAT denervated and ARH astrocyte hM3Dq-transduced mice with a single i.p. injection of J60 and extracted inguinal iWAT for western blots of total HSL and p-HSL two hours post the injection. Astrocyte stimulation failed to increase the phosphorylation of HSL on the denervated iWAT compared to sham-treated controls (Fig. 6d–f). This result suggests that ARH astrocytes modulate iWAT lipolysis by enhancing local neural inputs to the iWAT pad.

### Astrocyte stimulation excites POMC neurons in the ARH

**Fig. 7:**
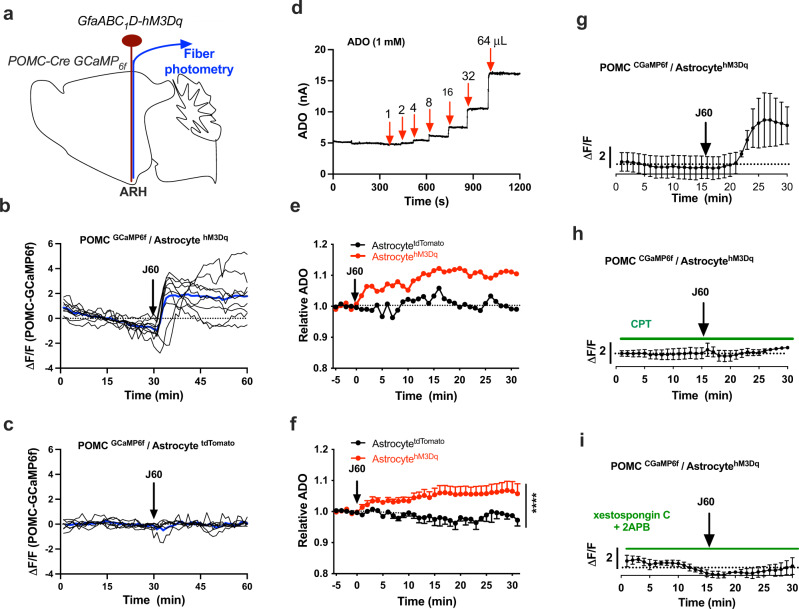
Astrocyte stimulation excites POMC neurons and elevated extracellular ADO. **a** Schematic illustration of targeting a vector carrying GfaABC_1_D-hM3Dq to ARH and implanting a photometry fiber over ARH in dual POMC-Cre GCaMP_6f_ mice. **b**, **c** Individual photometry traces showing that astrocyte stimulation with J60 via i.p. increased the intensity of POMC neuron GCaMP_6f_ signals in the astrocyte **b** hM3D-transduced mice (*n* = 8) compared to **c** tdTomato-transduced mice (*n* = 6). The blue line represents the average of the individual lines. **d** ADO probe calibration curve using increased volumes of ADO (1 mM) following the detailed instructions for the probes. **e** Representative traces of real-time recording of the signals of ADO in astrocyte hM3Dq or tdTomato-transduced mice. Mice were treated with J60 via i.p. after a stable baseline. **f** Averaged traces from **e** (tdTomato, *n* = 4; hM3Dq, *n* = 5). **g** Astrocyte stimulation with J60 addition in circulating aCSF increased POMC neuron GCaMP_6f_ signals (*n* = 4). **h** CPT blockade of ADORA_1_ diminished the effect of astrocyte stimulation with J60 (*n* = 4). **i** Pre-treatment of brain slices with IP_3_R blockers diminished the effect of astrocyte stimulation (*n* = 3). Two-way ANOVA with Tukey post hoc tests; data represent mean ± s.e.m.; ****p < 0.0001. Black arrows point to i.p. injections of J60 in **b**, **c**, **e**, **f**, and J60 addition in circulating aCSF in (**g**)–(**i)**.

We next sought to identify the involved sympathetic outflow neurons in the ARH. We performed real-time photometry monitoring of POMC neuron activity in dual POMC-Cre GCaMP_6f_ mice, in which POMC neurons were encoded with GCaMP_6f_ and astrocytes were virally transduced with the hM3Dq, and photometry fiber was implanted over the ARH (Fig. 7a). Astrocyte stimulation with a single J60 i.p. injection potently increased the intensity of POMC neuron GCaMP_6f_ signals in the astrocyte hM3Dq transduced mice (Fig. 7b) compared to controls (Fig. 7c). Ample evidence indicates that astrocyte stimulation elevates extracellular adenosine (ADO)^29–32^. We performed real-time monitoring of ADO using ADO probes in the virally transduced mice. A pre-calibrated ADO biosensor electrode (Fig. 7d) was inserted into ARH through a secured guide cannula in the astrocyte hM3Dq transduced mice. J60 administration with J60 via i.p. significantly increased the intensity of ADO signals in the astrocyte hM3Dq-transduced mice compared to control mice (Fig. 7e, f).

To examine whether ADO signaling is involved in the astrocyte excitation of POMC neurons, we performed photometry monitoring of POMC neurons by placing an optic fiber on the surface of brain slices from astrocyte hM3Dq-transduced dual POMC-Cre GCaMP_6f_ mice. J60 addition in the circuiting aCSFs increased the intensity of POMC neuron GCaMP_6f_ signals (Fig. 7g). Pre-incubation of brain slices with a selective ADO receptor type A_1_ (ADORA_1_) blocker 8-cyclopentyltheophylline (CPT) blocked the astrocyte effect (Fig. 7h). Activation of ADORA_1_ stimulates IP_3_-sensitive calcium stores^33^. To test whether astrocytic excitation of POMC neurons was caused by increased Ca^2+^ release from IP_3_-sensitive stores, we pretreated brain slices with two membrane-permeable IP_3_ receptors (IP_3_R) blockers xestospongin C and 2-aminoethoxydiphenyl-borate (2-APB). Inhibition of IP_3_R also blunted the astrocytic excitation of POMC neurons (Fig. 7i).

### POMC neurons partake in astrocytic regulations of iWAT sympathetic outflow and lipolysis

**Fig. 8:**
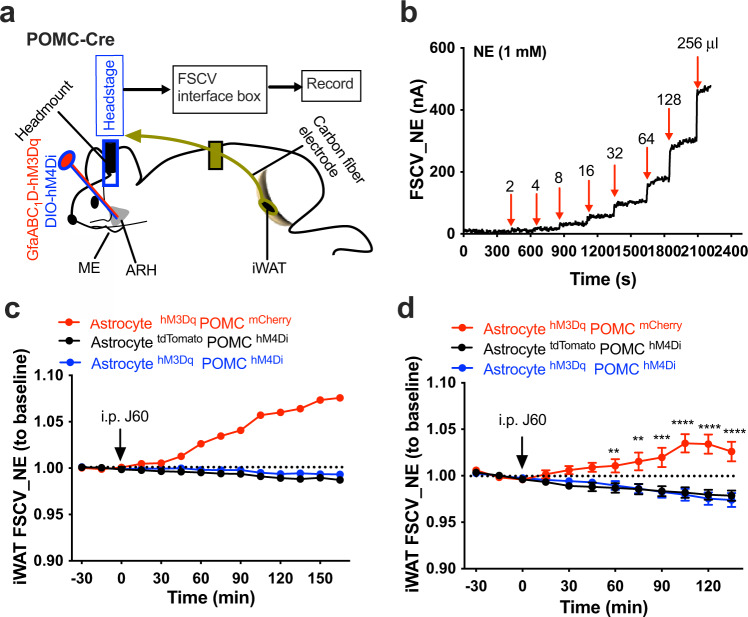
Inhibition of POMC neurons diminishes astrocytic elevation of iWAT NE contents. **a** Schematic illustration of targeting a vector carrying GfaABC_1_D-hM3Dq and a second vector carrying Cre-dependent hM4Di to the ARH and of implanting an FSCV carbon fiber electrode in iWAT in POMC-Cre mice. **b** NE probe calibration curve using increased volumes of NE (1 mM) following the detailed instructions for the probes. **c** Representative traces of real-time recording of the FSCV signals of iWAT in astrocyte hM3Dq POMC neuron mCherry (*n* = 5), astrocyte tdTomato POMC neuron hM4Di (*n* = 5), or astrocyte hM3Dq POMC neuron hM4Di (*n* = 6)-transduced mice, respectively. Mice were treated with J60 via i.p. after a stable baseline. **d** Averaged traces from **c** (Astrocyte^hM3Dq^ POMC^hM4Di^, *n* = 6; Astrocyte^hM3Dq^ POMC^mCherry^, *n* = 5; Astrocyte^tdTomato^ POMC^hM4Di^, *n* = 5). Two-way ANOVA with Sidak post hoc tests; data represent mean ± s.e.m.; **p = 0.006 < 0.01; ***p = 0.0002 < 0.001; ****p < 0.0001. Red arrows in **b** indicate the time points of adding NE in different volumes. Black arrows in **d** point to i.p. injections of J60.

To test it, we transduced ARH astrocytes with the stimulatory hM3Dq and POMC neurons with the inhibitory Cre-dependent hM4Di in POMC-Cre mice. Real-time monitoring of iWAT NE contents was performed using a fast scan cyclic voltammetry (FSCV) system, respectively, in the astrocyte hM3D POMC mCherry-, astrocyte hM3Dq POMC hM4Di, or astrocyte tdTomato POMC hM4Di-transduced POMC-Cre mice. The reason for using the FSCV method instead of the above-stated GCaMP_6f_ approach was that it currently is not feasible to perform selective Cre-dependent expressions in POMC neurons in dual TH-Cre GCaMP_6f_ mice or triple POMC-Cre TH-Cre GCaMP_6f_ mice. A pre-calibrated carbon fiber electrode connected to a head stage was implanted in iWAT via a guide cannula (Fig. 8a, b). After a stable baseline recording, J60 was administered via a single i.p. injection. Astrocyte stimulation increased adipose NE contents in the astrocyte hM3Dq POMC neuron mCherry-transduced mice but not in the POMC neuron hM4Di transduced mice (Fig. 8c, d). Inhibition of POMC neurons did not induce significant effects in the astrocyte tdTomato POMC neuron hM4Di-transduced mice (Fig. 8c, d), suggesting that POMC neurons do not modulate iWAT tonic sympathetic innervations. In cohort groups of the virally transduced mice, we evaluated the ability of POMC neurons to modulate local sympathetic inputs to the inguinal iWAT by performing electrophysiological recordings of the iWAT-supporting sympathetic nerves in lightly sedated mice. Consistently, chemogenetic stimulation of hM3Dq-transduced astrocytes with J60 via i.p. elevated the firing rates of sympathetic nerves innervating iWAT in astrocyte hM3Dq POMC neuron mCherry-transduced mice but failed in the POMC neuron hM4Di-transduced mice (Supplementary Fig. 8).

Next, we examined whether POMC neurons participated in the astrocyte stimulation-induced phosphorylation of the HSL. We transduced ARH astrocytes with hM3Dq and POMC neurons with hM4Di. Two hours post a single i.p. injection of J60, we extracted iWATs which were directly used for western blots of p-HSL, HSL, and actin. Astrocyte stimulation with J60 administration via i.p. increased the levels of phosphorylated HSL in the astrocyte hM3Dq POMC neuron mCherry-transduced mice compared to the astrocyte tdTomato POMC neuron mCherry-transduced controls but failed to increase the levels of p-HSL in astrocyte hM3Dq POMC neuron hM4Di-transduced mice (Supplementary Fig. 9a, b).

## Discussion

Precise control of adipose tissue sympathetic strength and adipocyte function is crucial for the regulation of adipose tissue lipid metabolism and body weight. By taking advantage of coordinated combined central and peripheral neurobiology methods and adipose tissue biochemical assays, our results in the current study provide the evidence that ARH astrocytes modulate sympathetic drive to iWAT, and reveal a previously unprecedented central astrocyte–peripheral white adipocyte axis promoting lipolysis. These findings fill in a missing but important gap in our understanding of non-neuronal glial mechanisms that orchestrate adipocyte functions. Meanwhile, our photometry approach measuring adipose tissue sympathetic NE contents and nerve activity would provide an alternative method to evaluate the dynamics of sympathetic strength in adipose tissues.

Previous studies^29,30,32^ and our findings in this study demonstrate that stimulation of astrocytes increases extracellular levels of ADO although the sources of ADO remain unclear. Pharmacological activation of central ADORA_1_ induces white adipose tissue lipid lipolysis, decreases body weight, and increases adipose NE contents^34^. Hypothalamic astrocytes express leptin receptors^35–37^ through which leptin elevates astrocytic Ca^2+^ levels^37^. The increased Ca^2+^ is required to induce Ca^2+^-dependent elevation of extracellular ADO^30,32^. ADO exerts bidirectional regulations of adipocyte functions through central ADORA_1_ signaling in the mammalian brains and peripheral tissues, respectively. For example, in vitro studies show that ADO induces adipogenesis by activating peripheral adipose ADORA_1_^38–40^, and the study by Gnad et al. (2014)^41^ indicates that ADO increased adipose lipolysis through activation of ADORA_2A_ in human adipocytes. This functional heterogeneity of ADO is probably attributable to different ADO receptor subtypes with unique pharmacological profiles and tissue distributions^42,43^ as well as specific physiological effects^44^. These findings, including ours, let us assume that astrocytes probably participate in the leptin regulations of sympathetic innervations to adipose tissues and adipocyte functions. Astrocytes may be stimulated by increased leptin during the early stage of high-fat diet (HFD) feeding and counter-regulate energy surfeit by promoting adipocyte functions such as lipolysis. Leptin would fail to stimulate astrocytes in leptin-resistant mice, which would subsequently tone down adipocyte functions with decreased lipolysis and increased fat deposition, causing a vicious circle of fat accumulation in HFD-fed mice. Pharmacogenetic stimulation of astrocytes and activation of ADORA_1_ signaling in the hypothalamus would provide an attractive therapeutic strategy to ameliorate leptin resistance-related metabolic symptoms.

The sympathetic outflow neurons partaking in the astrocyte effects remain unknown. There are different neuron populations in the ARH with different behavioral and metabolic functions. For example, agouti-related protein (AgRP) and POMC neurons are two functionally opposed neuronal populations intermingled in the ARH. Stimulation of AgRP neurons increases feeding^45^ and POMC neurons function to prevent body weight gain^46,47^. AgRP neurons project inhibitory GABAergic inputs to POMC neurons^48^. It also is meaningful to mention that the current study was focused to study the acute effects of astrocyte stimulation in satiated mice which were not likely caused by hunger. Astrocyte stimulation silenced AgRP neurons via the ADORA_1_ signaling pathway^49^. Our data in the current study showed that astrocyte stimulation excited POMC neurons and pharmacological inhibition of ADORA_1_ and IP_3_R-sensitive stores blunted this effect. These results suggest that astrocytic excitation of POMC neurons is probably attributable to disinhibition by POMC neuron-projecting inhibitory neurons such as AgRP neurons, or caused by direct ADORA_1_-G_i/o_ signaling-mediated Ca^2+^ release from IP_3_R stores in POMC neurons, which is probably supported by that activation of ADORA_1_ stimulates IP_3_-sensitive calcium stores in the forebrain neurons^33^. We, therefore, posited that astrocytes bidirectionally regulate AgRP and POMC neurons to directly or indirectly drive sympathetic premotor neurons to modulate sympathetic preganglionic and postganglionic neurons localized in the intermediolateral nucleus in the spinal cord.

Our findings in the current study show that astrocyte stimulation excites POMC neurons in the ARH via ADORA_1_ signaling and chemogenetic inhibition of POMC neurons and/or T_13_-L_1_ PG blunts the astrocyte effects on adipose tissues, suggesting that astrocytes modulate POMC neurons to orchestrate adipose tissue-innervating sympathetic preganglionic and postganglionic neurons through direct and/or indirect neural circuit projections. In addition to astrocytes, there also exist other types of cells including microglial cells. Our astrocyte selective vectors did not transduce microglia while we cannot exclude the possibility that they could be indirectly stimulated and potentially participate in the astrocyte effects on adipose tissues. It will also be of significance in developing microglia-selective approaches to define the roles of microglia in the regulation of adipocyte functions in the future, which would require many efforts beyond the scope of this study.

In the current study, we used cell-type specific manipulations and real-time photometry monitoring of iWAT sympathetic outflow neurons and nerve activity both in live mice and ex vivo tissues, which allowed us to acutely and accurately identify and manipulate the iWAT-supporting sympathetic ganglia. We provided functional evidence that both T_13_ and L_1_ PG provide sympathetic inputs to iWAT and partake in the astrocytic regulations of adipose sympathetic drive and adipocyte functions. We cannot exclude other neuron populations and ganglia as well as whether the astrocyte effects are iWAT specific or systemic. It is not technically feasible to target or manipulate those iBAT-supporting sympathetic ganglia (i.e., T_1_–T_5_) in live animals, which limits us to dissect and manipulate the brain–ganglia–iBAT circuits in live mice using the procedures in the current study. Our data show that astrocyte stimulation did not affect gene expressions of thermogenic markers, suggesting that acute astrocyte stimulation in the experimental conditions applied in this study was not sufficient to induce iWAT beiging and thermogenesis. This might be explained by that WAT beiging and BAT thermogenesis would require sustained and systemically integrated sympathetic innervations. Our experimental paradigm in the current study did not affect the total HSL protein and the genes encoding lipolytic enzymes, suggesting that our experimental protocol induces a rapid brain–adipose axis activity-dependent lipolysis.

Here, we propose an important central astrocyte–peripheral white adipocyte axis promoting adipose sympathetic outflows and adipocyte functions through POMC neurons and sympathetic PG, and the adipose sympathetic strength and adipocyte function scaling mediated by glial cells described here may be useful in the development of new therapeutic targets in obesity prevention and treatment.

## Methods

Experimental protocols were approved by the Institutional Animal Care and Use Committees at the Albert Einstein College of Medicine and conducted following the U.S. National Institutes of Health guidelines for animal research.

### Animals

C57/BL6J wild-type mice as well as Ai95(RCL-GCaMP_6f_)-D (Ai95D)^50^, TH-Cre^51^, and POMC-Cre^47^ transgenic mice have been described previously and are available from The Jackson Laboratory. Both male and female mice (age 8–12 weeks) were used at the start of experiments. Mice were group-housed 3–5 mice per cage in temperature (22–25 °C)- and humidity-controlled housing rooms on a 12-h light:12-h dark cycle, with lights on from 8:00 a.m. to 8:00 p.m., and with ad libitum access to water and mouse regular chow (PicoLab Rodent Diet 20, 5058, LabDiet). The virally transduced or implanted mice were allowed to recover on a heating pad or a heat lamp and we kept close observations until they fully recovered from the anesthesia. We housed them individually and gave them one Meloxicam MD tab per day for 3 days. We monitored the animals daily for potential infection or other signs of adverse reactions such as lethargy and weight loss. We applied antibiotic ointment if we observed minor skin infections. We contacted veterinary staff for treatment if we observed more severe infections. The animals were euthanized if they were lethargic and lost more than 15% of body weight.

### Pharmacology

All the chemicals were purchased from Sigma except for J60 purchased from HelloBio. For experiments requiring intraperitoneal injections (i.p.), we used 27-gauge needles, and the stocks of the chemicals were diluted in the vehicle (200 μl) at the final dose of J60 (1 mg/kg) on the experimental days. For acute brain slice physiology and photometry studies, the final doses of J60 (10 μM) and CTP (1 μM) were present in the circulating solutions. For those experiments with brain slices pre-treated with Xestospongin C and 2-APB, brain slices were pretreated with the Xestospongin C (1 μM) and 2-APB (100 μM) for 1–3 h, and the chemicals were kept in the circulating solutions during the whole experiments.

### Vectors

Vectors used in this study included: AAV vectors for astrocyte expressions of hM3Dq (AAV_5_-GfaABC_1_D-hM3Dq-mCherry, Addgene#92284, titer at 4.1 × 10^13^ vg/ml), GCaMP_6f_ (AAV_5_-GfaABC_1_D-cyto-GCaMP_6f_, Addgene#52925, titer at 7.0 × 10^12^ vg/ml), and ChR2 (AAV_5_-GFAP104-ChR2-mCherry, Addgene#58892, titter at 3.4 × 10^13^ vg/ml). AAV retrograde vectors for adipose nerve expressions of GRAB_NE (AAV_rg_-hSyn-GRAB_NE1m, Addgene#123308-AAVrg, titer at 7 × 10^12^ vg/ml) and Cre-dependent hM3Dq (AAV_rg_-hSyn-DIO-hM3Dq-mCherry, Addgene#44361-AAVrg, titer at 7 × 10^12^ vg/ml). AAV vectors for PG injections: Cre-dependent ChR2 (AAV_2_-EF1a-DIO-hChR2-mCherry, Addgene#20297, titer at 1.6 × 10^12^ vg/ml) and hM4Di (AAV_2_-hSyn-DIO-hM4Di-mCherry, Addgene#44362, titer at 5.0 × 10^12^ vg/ml). Control vectors include AAV_5_-GfaABC_1_D-tdTomato (Addgene#44332, titer at 7.0 × 10^12^ vg/ml) and AAV_5_-hSyn-DIO-mCherry (Addgene#50459, titer at 1.5 × 10^13^ vg/ml). Animals transduced with control vectors were referred to as controls in the text. Viral vectors were aliquoted on arrival and stored at ∼80 °C prior to stereotaxic injections.

### Brain stereotaxic viral injections and fiber implantations

Mice were deeply anesthetized with isoflurane (3%) and placed in a stereotaxic frame (David Kopf Instruments, Tujunga, CA; or Harvard Apparatus, Holliston, MA). Mouse skulls were exposed via a small incision, and two small holes were drilled directly above the viral injection sites bilaterally on each side of the midline using a micro-precision drill (David Kopf Instruments; CellPoint Scientific). A pulled-glass pipette with 20–40 µm tip diameter was inserted into each side of the brain, and two injections (∼100 nl on each side) of the viral vectors were delivered into the ARH at coordinates (bregma: −1.46 mm; midline: ±0.2 mm; skull surface: −5.95 to −5.75 mm). A micromanipulator (Narishige) was used to control the viral injection at a speed of 30 nl per min, and the injection pipette was withdrawn 15 min after the final injection to assure adequate viral delivery. For fiber photometry experiments in the ARH, an optic fiber ferrule (400 μm core, 0.48 numeric aperture; 1.25 mm diameter; Doric, MF1.25, 400/430-0.48, FLT) was inserted over the ARH at coordinates (bregma: −1.46 mm; midline: 0.2 mm; ventral: −5.5 mm). Grip cement (DENTSPLY) was used to anchor the ferrules and guide the cannula to the skull. Optic fiber dust caps were placed on optic fibers to keep the optic fibers clean. Mice were returned to their home cages and singly housed typically for at least two weeks for recovery and viral expression before performing the experiments.

### IWAT viral injections and fiber implantations

Following published protocols^22^ with minor modifications, mice were anesthetized with isoflurane (3%) delivered by a SomnoSuite anesthesia system (Kent Scientific) through a face mask and placed in a surgical platform (Kent Scientific). The back over the iWAT pad was shaved and wiped with 10% povidone–iodine solution and then 70% ethanol three times, and an incision (2 cm) along the dorsal–ventral axis of the iWAT pad was made to expose the dorsolumbar iWAT from surrounding tissues. The lymph node at the junction between dorsolumbar and inguinal portions of iWAT was used as an anatomical marker. We took care not to damage underlying neural and vascular supplies to the pad. A pulled capillary needle with 20 µm tip diameter was inserted into the iWAT, and 10 injections of 10 μl of viral vectors were made under a stereo microscope (AmScope). At each site, we delivered 1 μl of the vectors into the pad (∼4 mm deep) and left the needle in place for ∼1 min to assure adequate delivery. A micromanipulator (Narishige) was used to control the viral injections and the injection pipette was withdrawn 1 min after each injection. For the iWAT fiber photometry experiments, a sleeve-connected conventional optic ferrule (400 μm core, 0.48 numeric aperture; 1.25 mm diameter; 5.5 mm length; Doric, MF1.25, 400/430-0.48, FLT; Doric) for tethered photometry was inserted inside the iWAT along the incision, and optic fiber dust caps were placed on optic fibers to keep the optic fibers clean. For wireless photometry experiments, a sleeve-connected TeleFipho fiber-optic cannula (400 μm core, 0.39 numeric aperture, 425 μm cladding, 2.5 mm diameter, and 5.5 mm length; Amuza Inc) was inserted into the injected iWAT along the incision. The antibacterial powder was applied under the skin before closing the incision with a surgical suture. To further stabilize the implanted fibers or cannula, we wrapped the incisions as well as the fibers and cannular with a self-adhesive elastic wrap (1-inch width, 3M). To shield room light, we covered the wrap with a light shielding tape 603HL (3M).

### Two-channel dual-wavelength fiber photometry (FP)

Two excitation wavelengths were used: 465 and 405 nm. The wavelength of 465 nm excites calcium-dependent fluorescence from GCaMP protein. Excitation light intensities were modulated at frequencies (208.62 and 572.21 Hz for 405 and 465 nm, respectively) to avoid contamination from overhead lights (120 Hz and harmonics) and cross-talk between excitation lights. Excitation lights were generated through fiber-coupled two connectorized LEDs (CLED_465 for 465 nm with output at 21.4 mW for fiber 400 μm NA = 0.53; CLED_405 for 405 nm with output at 21 mW for fiber 400 μm NA = 0.53; Doric Lenses) driven by a two-channel LED driver (LEDD_2; Doric Lenses). The LEDD_2 was controlled by a fiber photometry console (FPC; Doric Lenses) connected to a computer. Excitation lights were passed through fluorescence MiniCube (B340-1217; Doric Lenses; iFMC4_AE (405)E(460–490)_F(500–550)_S) integrated with Fluorescence Detector Head allowing for an increase in signal transmission. The single detector measures both signals within the fluorescence detection window from 500 to 550 nm band. The combined excitation light was sent into a patch cord made of a 400 µm core, 0.48 NA, low-fluorescence optical fiber (Doric Lenses; MFP_400/460/1100-0.48_1m_FCM-MF1.25). The patch cord was connected to an implanted fiber contained in a 1.25 mm diameter ferrule via a sleeve (Doric Lenses; Zirconia Sleeve 1.25 mm with black cover; Sleeve_ZR_1.25-BK). The GCaMP emission fluorescence signals were collected through the same patch cord and passed through the same Minicube and focused onto a Fluorescence Detector Head (FDH; Doric Lenses). The FP experiments were run in a Lock-in mode, and the acquisition rate was set to 12.0 ksps*C controlled by Doric Neuroscience Studio software (V5.3.3.14). The FP experiments were performed 5 min after connecting the optic fibers to the animals. To achieve maximum sensitivity and avoid saturating the detector, the *V*_max_ was set to 0.6 and 1.2 V, respectively, for analog#1 and analog#2, and the *V*_min_ was set to 0.1 V for both, and the fluorescence detector amplifier (FDA; Doric Lenses) was set to 10X in AC mode. At the tip of the fibers, the 465 nm signal was set to 20–26 µW, and the 405 nm signal was set to 7–10 µW.

### Wireless fiber photometry (TeleFipho)

For experiments performing TeleFipho recordings, the TeleFipho system (Amuza Inc) includes a small and light-weighted headstage and wireless transmission circuit. The TeleFipho headstage (3 g weight; 12 × 12 × 22 mm dimensions) is composed of a filter cub, photodetector, and blue light source (excitation wavelength peaked at 470 nm with 445–490 nm filter band; emission wavelength with a 500–550 nm band). The sampling rate was set at 100 Hz with a gain of 10^10^ V/A. The signal transmission distance was within 2 m. The receiver containing a 1× photometry analog out and 1× general-purpose analog were connected to a PC installed with the TeleFipho software. The light power was adjusted to reach a baseline of around 35,000 following the software instructions to achieve maximum sensitivity and avoid saturating the detector. The maximum power of the 465 nm was 300 µW. We normalized the GCaMP_6s_ signal intensity (I) for each trial and calculated the *Z* score as (*I*−*I*_mean_)/*I*_SD_, where *I*_mean_ and *I*_SD_ represent the mean and standard deviation of the signals for each mouse. The TeleFipho experiments were performed 5 min after placing the head stage.

### Fiber photometry and photostimulation in acute ex vivo ganglia and tissues

Acute ganglia and tissues were dissected rapidly and placed in ice-cold oxygenated (95% O_2_ and 5% CO_2_) solution containing the following (in mM): 110 choline chloride, 2.5 KCl, 1.25 mM NaH_2_PO_4_, 2 CaCl_2_, 7 MgSO_4_, 25 d-glucose, 3.1 Na-pyruvate, and 11.6 Na-l-ascorbate, pH 7.3. The dissected ex vivo ganglia and tissues were maintained in an incubation chamber at 34 °C for 30 min and then brought to room temperature until transferred to a recording chamber. During the experiments, ganglion or tissue was transferred to a submersion-recording chamber in an isolated table (TMC) and continuously perfused with the recording solution containing (in mM): 119 NaCl, 25 NaHCO_3_, 11 d-glucose, 2.5 KCl, 1.25 MgCl_2_, 2 CaCl_2_, and 1.25 NaH_2_PO_4_, aerated with 95% O_2_/5% CO_2_ (1–2 ml/min at 30–35 °C). Optic fibers for photostimulation and/or photometry or (400 μm NA = 0.57; Doric Lenses) were secured and controlled by a Dual micromanipulator system for patch clamp (Sensapex) and placed on ganglion and tissues for photostimulation and photometry, respectively, 15–30 min after placing the optic fibers. Photostimulation was detailed in the text and figure legends.

### Anesthetized fiber photometry monitoring of inguinal iWAT neural inputs

To perform fiber photometry monitoring of the TH-positive neural inputs in inguinal iWAT in dual TH-Cre GCaMP_6f_ mice, the ARH astrocyte ChR2-transduced mice were maintained under a light plane of anesthesia (∼1% isoflurane) with a low-flow anesthesia system (SomnoSuite) equipped with MouseSTAT temperature and heart monitor modules (Kent Scientific Co.). At this anesthesia level, a toe pinch reflex was elicited throughout the whole experiment. The iWAT was exposed and the nerve innervating iWAT was isolated without damaging surrounding blood vessels. A photometry fiber was placed on the entry site of the ACFN in the iWAT.

### In vivo adipose sympathetic nerve electrophysiological recordings

Mice were anesthetized with isoflurane (3%) delivered by a SomnoSuite anesthesia system (Kent Scientific) through a face mask and placed on a warm pad (∼28 °C) in a surgical platform (Kent Scientific) on a vibration-isolated table (TMC). The temperature was maintained using a Heat Therapy Pump (HTP-1500). An incision (2 cm) along the dorsal–ventral axis of the iWAT was made to expose the iWAT pad from surrounding tissues. We took care to isolate the iWAT-supporting anterior cutaneous femoral nerve (ACFN) branch, a visible nerve under a stereomicroscope, and severed distal to the electrode placement site to disconnect afferent fibers, and the efferent (proximal) end of the nerve was placed on two coated silver hook electrodes (254 μm diameter, A-M Systems). A mineral oil/silicone elastomer (Kwik-Cast, WPI) mixture (1:1) was applied to the exposed site to completely surround the electrode/nerve connections to insulate electrical noise and reduce tissue drying. Mice were then maintained under a light plane of anesthesia. A stable anesthetic state was maintained during the whole experiment. The recording electrodes were held and placed using a micromanipulator (SENSAPEX), and the nerve firing signals were recorded in an IC mode using MultiClamp amplifier and analyzed using pClamp 11.0 (Molecular Devices). The primary and secondary outputs were set at 100× AC membrane potential. We visualized and audibled the analog signals using an external oscilloscope connected to the amplifier. For the detection of the recorded signals, we used template matching (Clampfit, Molecular Devices) with a set threshold followed by visual inspection. We analyzed the signals at 5-min bins and normalized them to the basal signals.

#### Real-time monitoring of adipose NE contents by using fast-scan cyclic voltammetry

Following the surgical procedures as described above, BASi mouse guide cannulas (5 mm length and 600 μm diameter; Pinnacle#7032) were stereotaxically implanted inside the iWAT. Adipose tissue NE contents were detected using a modified tethered FSCV mouse system (Pinnacle). Mouse carbon fiber electrodes (7004-CFE; Pinnacle) were inserted in previously implanted BASi mouse cannula inside iWAT with an extension of sensing tip (0.5 mm length; 34 μm diameter). The electrodes were connected to the FSCV head-stage via a 4-channel mouse commutator/swivel using a custom bypass cable. The head stage was connected to an FSCV interface box. An Ag/AgCl reference electrode was implanted under animal skin and also connected to a head stage secured on the mouse head. Following the detailed procedures as described in the Pinnacle FSCV software (2.0.9), a voltage span (−0.6 to +1.5 V) was applied to detect NE contents by using the preprogrammed triangle waveform for NE detection. The electrical signals were recorded with 250 cycles per min and 250 ms per sweep in freely moving animals placed in home cages and transmitted to a computer and analyzed using the FSCV software (Pinnacle). The CFE electrodes were calibrated before and after the measurements in phosphate-buffered saline with the addition of 10 μm NE increments.

### Photometry monitoring of POMC neuron activity

ARH POMC neuron GCaMP_6f_ and astrocyte hM3Dq-transduced mice were deeply anesthetized with isoflurane and decapitated. Mouse brains were dissected rapidly and placed in ice-cold oxygenated (95% O_2_ and 5% CO_2_) solutions containing the following (in mM): 110 choline chloride, 2.5 KCl, 1.25 NaH_2_PO_4_, 2 CaCl_2_, 7 MgSO_4_, 25 d-glucose, 3.1 Na-pyruvate, and 11.6 Na-l-ascorbate, pH 7.3. Coronal brain slices containing the ARH (300 μm thick) were cut with a vibratome (Leica VT 1200S) and maintained in an incubation chamber (34 °C) for 30 min, and then brought to room temperature until transferred to a recording chamber. During the experiments, the slice was transferred to a submersion-recording chamber in an isolated table (TMC) and was continuously perfused with the recording solution containing (in mM): 119 NaCl, 25 NaHCO_3_, 11 d-glucose, 2.5 KCl, 1.25 MgCl_2_, 2 CaCl_2_, and 1.25 NaH_2_PO_4_, aerated with 95% O_2_/5% CO_2_ (1–2 ml/min at ∼30 °C). A nylon mesh was used to hold a piece of the slice in place in the circulating solution in the recording chamber. An optic fiber (400 μm NA = 0.57; Doric Lenses) was placed on the ARH for photometry monitoring Ca^2+^-dependent GCaMP_6f_ signals in POMC neurons. Real-time photometry monitoring was started 15–30 min after placing the optic fibers. Chemicals or vehicles were added to the circulating solutions as detailed in the text and figure legends. For the experiments requiring preincubations, brain slices were pre-incubated with the chemicals for 1–3 h before transferring to the recording chamber and the chemicals were in the circulating solution during the whole experiment.

### In vivo real-time measurements of extracellular adenosine in the ARH

A BASi mouse guide cannula (5 mm length and 600 μm diameter; Pinnacle#7032) was stereotaxically implanted over ARH (bregma: −1.46 mm; midline: 0.2 mm). After two weeks of recovery from the surgery, a mouse biosensor with integrated reference (Pinnacle#7004) coated with adenosine enzymatic matrix surrounding the sensing cavity (Sarissa, UK/Pinnacle, USA) was inserted in the implanted guide cannula with an extension of sensing cavities (1.0 mm length and 176 μm diameter). A voltage (+0.6 V) was applied to the sensing electrodes generated from a potentiostat (Model 8102; Pinnacle Technology, Inc, US). The electrical signals were recorded in 1-s bins in mice placed in home cages and transmitted to a computer and analyzed using the Pinnacle Sirenia Acquisition software (version 2.2.5). Adenosine biosensors were calibrated before and after the in vivo measurements in phosphate-buffered saline with the addition of 10 μm adenosine increments.

### Inguinal iWAT tissue western blot

The freshly extracted inguinal iWATs were immediately frozen in liquid nitrogen. The frozen samples were stored at −80 °C freezer or kept on ice for immediate homogenization. Briefly, an 800 μl ice-cold lysis buffer was added to the tube for 100 mg of tissue from each animal, and the tubes were vortexed on ice for 2 h. The tubes were then centrifuged for 30 min at 14,000 rpm at 4 °C in a microcentrifuge. The supernatants were aspirated and transferred to a fresh tube on ice. The primary rabbit anti-phospho-HSL(Ser660) (PA5-64494; Invitrogen) and rabbit anti-HSL (PA5-17196; Invitrogen) antibodies were diluted at 1:1000. The protein expressions were detected by using traditional western blots or automated complete protein analysis solution (Jess; protein simple). For traditional western blots, the extracted protein was loaded in 10% SDS–PAGE gel at a voltage of 100–200 V for 1.5 h. Proteins were transferred to the PVDF membrane at 100 V for 1 h. The transferred PVDF membrane was then blocked in blocking buffer (5% milk, 0.1% Tween 20 in PBS) for 1 h and blotted with primary antibodies. Following three washes of PBS-T (0.1% Tween 20 in 1X PBS), IRDye 800 CW donkey anti-rabbit IgG secondary antibody (#926-32213; Li-Cor) dissolved in PBS-T (1:10,000) was applied for 1 h at room temperature. The blots were washed three times with the PBST and imaged infrared imaging system (Odyssey). The secondary antibodies used for Jess included an anti-rabbit secondary NIR antibody (043-819; ProteinSimple) and an anti-mouse secondary IR antibody (043-822; ProteinSimple), which were commercially designed not to dilute, following the instructions.

### Inguinal iWAT glycerol and plasma NEFA measurements

IWAT tissue glycerol was detected using an adipose tissue explant lipolysis assay kit (LIP-6-NC; Zenbio) following the detailed instructions in the kit. All tissue samples were normalized to total protein concentrations. For plasma NEFA assays, retro-orbital blood collections were performed from mice receiving different treatments as described in the text and figure legends. Blood samples were collected in EDTA-heparin coated tubes (SARSTEDT, Germany) and centrifugated at 4 °C for 15 min. Blood plasma was then collected and aliquoted at 50 μl/tube in −80 °C freezer. Plasma NEFA was detected with a free fatty acid kit (STA-618; Cell Biolabs, Inc.) following the instructions of the kit.

### Inguinal iWAT sympathetic nerve terminal stain

Following published protocols^5,12^ with some modifications, mice were deeply anesthetized and transcardially perfused with PBS containing 10 μg/ml heparin. Inguinal iWATs were extracted and fixed in 1x PBS containing 1% PFA and 10% sucrose at 4 °C overnight. The fixed iWATs were washed three times for 1 h using 1x PBS, and the connective tissues were removed under microscopy. About 2 mm^3^ of the fixed and cleaned pads were dehydrated at room temperature in the order of 20% methanol (30 min), 40% methanol (30 min), 60% methanol (30 min), 80% methanol (30 min), and 100% methanol (30 min) twice; and then bleached in 5% H_2_O_2_ in methanol containing 10 mM EDTA–Na (pH 8.0) at 4 °C for 48 h. The tissues were subsequently treated in 80% methanol (30 min), 60% methanol (30 min), 40 % methanol (30 min), 20 % methanol (30 min), and 1x PBS containing 0.2% Triton X-100 for 1 h twice. The tissues were permeabilized with 1x PBS containing 0.2% Triton X-100 and 20% DMSO and 0.3 M glycine for 24 h at 37 °C and then blocked using a blocking buffer containing 0.2% Triton X-100 and 20% DMSO and 5% donkey serum in 1x PBS at 37 °C for 24 h. Tissues were incubated with mouse monoclonal Alexa Fluor 647 (sc-25269-AF647; Santa Cruz)- or rabbit polyclonal Alexa Fluor 350 (bs-0016R-350; Bioss)-conjugated anti-TH antibodies diluted (1:150) in PBST (0.2% Tween-20, 10 μg/ml heparin, 5% DMSO, 5% donkey serum) at 37 °C for 72 h and protected from light. After washing three times with 1x PBS containing 0.2% Tween-20 and 10 μg/ml heparin at 37 °C with rotation for 2 h, the tissues were dehydrated at room temperature in methanol in the order as below: 20% (60 min), 40% (60 min), 60% (60 min), 80% (60 min), and 100% (60 min) twice; and then incubated in dichloromethane/methanol (2:1 in volume) overnight at room temperature and in 100% dichloromethane for 15 min twice; and cleared using 100% dibenzyl-ether for 1 h twice at room temperature. The transparent tissues were transferred into the well on the Press-to-Seal silicone isolator with adhesive (Invitrogen; P24744) and immersed with 100% dibenzyl-ether and covered by a cover slip for imaging. The tissues were imaged using a Leica SP8 Confocal microscopy at ×10 objective magnification with a step-size of 5 μm through the tissue block.

### Paravertebral ganglia (PG) sympathectomy and viral injections

Following previously documented procedures^27,28^, we made a ventrodorsal incision over the left kidney and pushed it down to expose the ganglia at thoracic vertebral level 13 (T_13_) and lumbar vertebral level 1 (L_1_). We excised both T_13_ and L_1_ ganglia by decentralizing them and cutting relevant white ramus and gray ramus. We took care not to damage the dorsal ramus and ventral ramus of the spinal nerves, blood vessels, and the dorsal root ganglia (DRG). We performed Sham control surgeries with similar procedures but no ganglia extraction and nerve cutting. For the PG viral injection experiments, we targeted a small volume of (50 nl) of vectors to the PG with a micromanipulator-controlled tiny capillary needle (20 μm in diameter) and left the needle for 5 min to assure adequate delivery without leaking. After the surgeries or viral injections, we gently replaced the kidney before closing the wound and applied antibacterial powder, and closed the incisions with surgical sutures.

### Lateral cutaneous femoral nerve (LCFN) and anterior cutaneous femoral nerve (ACFN) physical denervation

Mice were deeply anesthetized. An incision was cut along the inguinal portion of iWAT and the LCFN and ACFN were exposed. Under a stereoscope, we carried out a local neural crush injury to the inguinal iWAT-supporting LCFN and ACFN branches with forceps about 2 mm from their distal iWAT entry points, respectively. We performed Sham control surgeries with similar procedures but no nerve-crushing. After the surgeries, we applied antibacterial powder on the surface of iWAT and closed the incision with surgical sutures.

### Paravertebral ganglia (PG) stain

Dual TH-Cre GCaMP_6f_ mice were deeply anesthetized and transcardially perfused with 1× PBS (pH 7.4 containing 10 μg/ml heparin followed by 4% paraformaldehyde (PFA) in PBS. The PG chain was dissected and fixed overnight in 4% PFA and then in 10% sucrose for 3 days. We placed and embed the T_13_ PG with the cryo-embedding media OCT in a base mold. We sectioned 10 μm-thick cryostat sections using a cryotome (Leica CM1950) and mounted them on superfrost plus slides. The ganglia chain and sections were rinsed in 300 ml of 0.1% triton X-100 in PBS (0.1% PBS-TX) three times (10 min each time), incubated in PBS containing 3% H_2_O_2_ and 10% methanol at room temperature for 2 h, and rinsed again in 300 ml of 0.1% PBS-TX for three times (10 min each time). They were then permeabilized in 1% PBS-TX for 40 min at room temperature, dried, and incubated with mouse monoclonal Alexa Fluor 647 (sc-25269-AF647, Santa Cruz)- or rabbit polyclonal Alexa Fluor 350 (bs-0016R-350, Bioss)-conjugated anti-TH antibodies diluted (1:150) in 100 μl PBST at 4 °C overnight. We washed the tissues or sections 10 min three times in 300 ml 0.1 % PBS-TX and mounted them on glass slides for imaging.

### Brain histology and immunofluorescence

Mouse brains were fixed overnight in 4% PFA and then in 10% sucrose for three days. Mouse brain sections (40 μm) were cut using Vibratome (VT 1200S; Leica). Sections were washed 10 min with 0.1% PBS-TX three times and permeabilized for 30 min with 1% PBS-TX. The sections were blocked for 2 h with 1% BSA in 0.1% PBS-TX. Brain sections were incubated with Alexa fluor 488-conjugated rabbit anti-GFAP antibodies (bs-0199R-A488; Bioss), Alexa fluor 488-conjugated mouse anti-NSE antibodies (bsm-33072M-A488; Bioss), or Alexa fluor 350 conjugated rabbit anti-CD68 antibodies (bs-0649R-A350; Bioss) in a dilution of 1:150 at 4 °C for 24−48 h. Brain sections were then washed 10 min with 0.1% PBS-TX three times, mounted on glass slides, and cover-slipped for imaging using confocal microscopy (Zeiss SP8). The images were analyzed using Leica Application Suite X (LAS X). To validate our viral transduction efficiency, we performed post hoc examinations of the transduced astrocytes. As stated above, we perfused and sectioned the brains (40 µm thick) of the hypothalamus. We took images of the sections and manually counted the astrocytes labeled by the virus-tagged fluorescent proteins (Supplementary Fig. 10).

### Total RNA isolation

Inguinal iWATs were extracted from mice. Total RNA was isolated from extracted tissues using TRIZOL (Invitrogen). Briefly, 50–100 mg fresh tissue was homogenized with 1 ml TRIZOL and incubated for 5 min at room temperature for the complete dissociation of nucleoprotein complexes. Chloroform (200 μl) per 1 ml of TRIZOL Reagent was added into the reaction tube. The reaction tube was vigorously shaken by hand for 15 s, incubated at room temperature for 2 min, and then centrifuged at 12,000 × *g* at 4 °C for 10 min. The aqueous phase was transferred to a new RNase-free tube in which an equal volume of isopropyl alcohol was added, kept at −20 °C for at least 20 min, and centrifuged at 12,000 × *g* for 10 min. The total RNA was washed with 70% alcohol, and then centrifuged for 15 min at no more than 7000×*g* at 4 °C. Reactions were run with M-MLV RT according to the manufacturer’s protocol (Roche). cDNA was quantified using the QuantStudio 3 Real-Time PCR system.

### Real-time quantitative PCR analysis (RT-qPCR)

RT-qPCR was performed using SYBR Green I (Roche) in a reaction volume of 20 μl in the QuantStudio 3 real-time PCR system (ThermoFisher). Reactions were performed using the LightCycler 480 (Roche) under the following procedure: 19 μl qPCR reaction mix (10 μl of 2x QuantTect SYBR Green I mix; 1 μl of 10 μM forward primer; 1 μl of 10 μM reverse primer; and 7 μl RNase free water) was added to 1 μl diluted cDNA using 96-well transfer plate (Roche). The PCR conditions were run as follows: activation at 94 °C for 10 min; 45 cycles of denaturation at 94 °C for 10 s, 60 °C for 20 s, and 72 °C for 20 s. The qPCR data were analyzed using the comparative Ct (2−∆∆Ct) method. The primer sequences for qRT-PCR are shown in Table 1.

**Table 1:** The primer sequences for qRT-PCR

Gene name	Sequence 5′−3′
Dio2 forward	AATTATGCCTCGGAGAAGACCG
Dio2 reverse	GGCAGTTGCCTAGTGAAAGGT
HSL forward	CGCCATAGACCCAGAGTT
HSL reverse	TCCCGTAGGTCATAGGAGAT
ATGL forward	CCAACACCAGCATCCAGT
ATGL reverse	CAGCGGCAGAGTATAGGG
Prdm16 forward	CCA CCA GCG AGG ACT TCA C
Prdm16 reverse	GGA GGA CTC TCG TAG CTC GAA
Ucp1 forward	GTGAACCCGACAACTTCCGAA
Ucp1 reverse	TGAAACTCCGGCTGAGAAGAT
Actin forward	GCAGGAGTACGATGAGTCCG
Actin reverse	ACGCAGCTCAGTAACAGTCC

### Statistics and reproducibility

Animals were randomly assigned to experimental or control groups. Following post hoc histological conformation of viral transfections and cannula/fiber placements, all mice with inaccurate viral infections or cannula placements were excluded from behavior experiments. Only mice with accurate viral injections and cannula placements were included in the data analysis. Success rates for viral injections varied for each individual experiment with about 80% of injected animals displaying accurate viral injections and/or cannula placement. All experiments were repeated at least twice for each mouse and average values were calculated for each individual mouse for statistical analysis. Mice were habituated to vehicle i.p. injection and related experimental procedures for 3–7 days before performing the tests. Paired or unpaired Student’s *t*-tests were used to analyze differences between two groups of the same or different mice when appropriate, respectively. One-way ANOVA with Tukey’s post hoc test was used to compare group data from more than two groups of mice. Repeated measures (RM) two-way ANOVA with the within-subject factors of time segment and treatment (vehicle vs. chemicals) or mixed ANOVA with the within-subject factor of time segment and the between-subjects factor of viral injections type were used to analyze data from more than two groups across various time points. Sidak’s post hoc test was used to test for significant effects at various time segments following the detection of a significant effect’s main effect or interaction. All data were analyzed by using Prism 9.0 (GraphPad Software).

### Reporting summary

Further information on research design is available in the Nature Portfolio Reporting Summary linked to this article.

## Supplementary information

Supplementary Information

Reporting Summary

## Data Availability

All the data generated and analyzed that support the findings in this study are within the article and its supplementary information files and are available from the corresponding author without restrictions. Source data are provided with this paper.

## Source data

Source Data
