# Supplementary material for: Deconstruction of a hypothalamic astrocyte-white adipocyte sympathetic axis that regulates lipolysis in mice
Source: Nat Commun. 2022 Dec 7;13:7536. doi: 10.1038/s41467-022-35258-6 (PMC9729228; doi:10.1038/s41467-022-35258-6)

Deconstruction of a hypothalamic astrocyte-white adipocyte axis for lipolysis

Dan Chen[1,2+], Yong Qi[2+], Jia Zhang[1,2], & Yunlei Yang[1,3,4,5*]

Supplementary Fig. 1 Neurons were not transduced by GfaABC$_1$D-dependent vectors. Correlated to the text Figure 1, representative images of Alexa Fluor 488-conjugated anti-NSE antibody labeled neurons (green) and virally mCherry-transduced cells (red). No colocalizations of A488-labeld NSE neurons and mCherry-transduced astrocytes were observed. Scale bar, 20 µm.

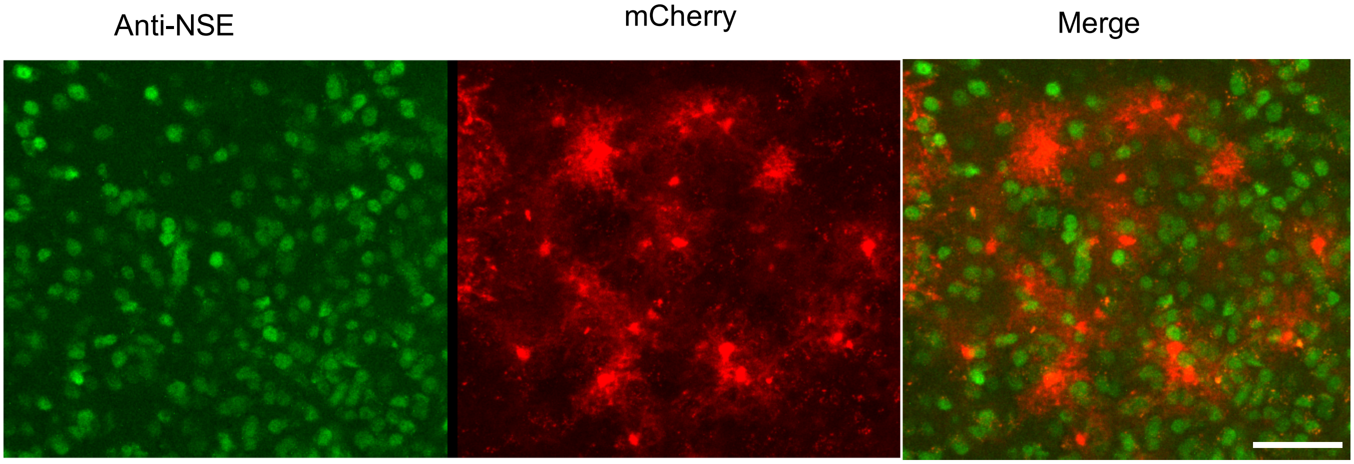

Supplementary Fig. 2 Microglia were not transduced by GfaABC$_1$D-dependent vectors. Correlated to the text Figure 1, representative images of Alexa fluor 488 anti-GFAP antibody-labelled cells (green), virally transduced mCherry-labelled cells (red), Alexa fluor 350 CD68 anti-CD68 antibody-labelled cells (blue) and colocalizations of the colors. No colocalizations of A350-labeld microglia and GFAP and mCherry-labeled cells were observed.

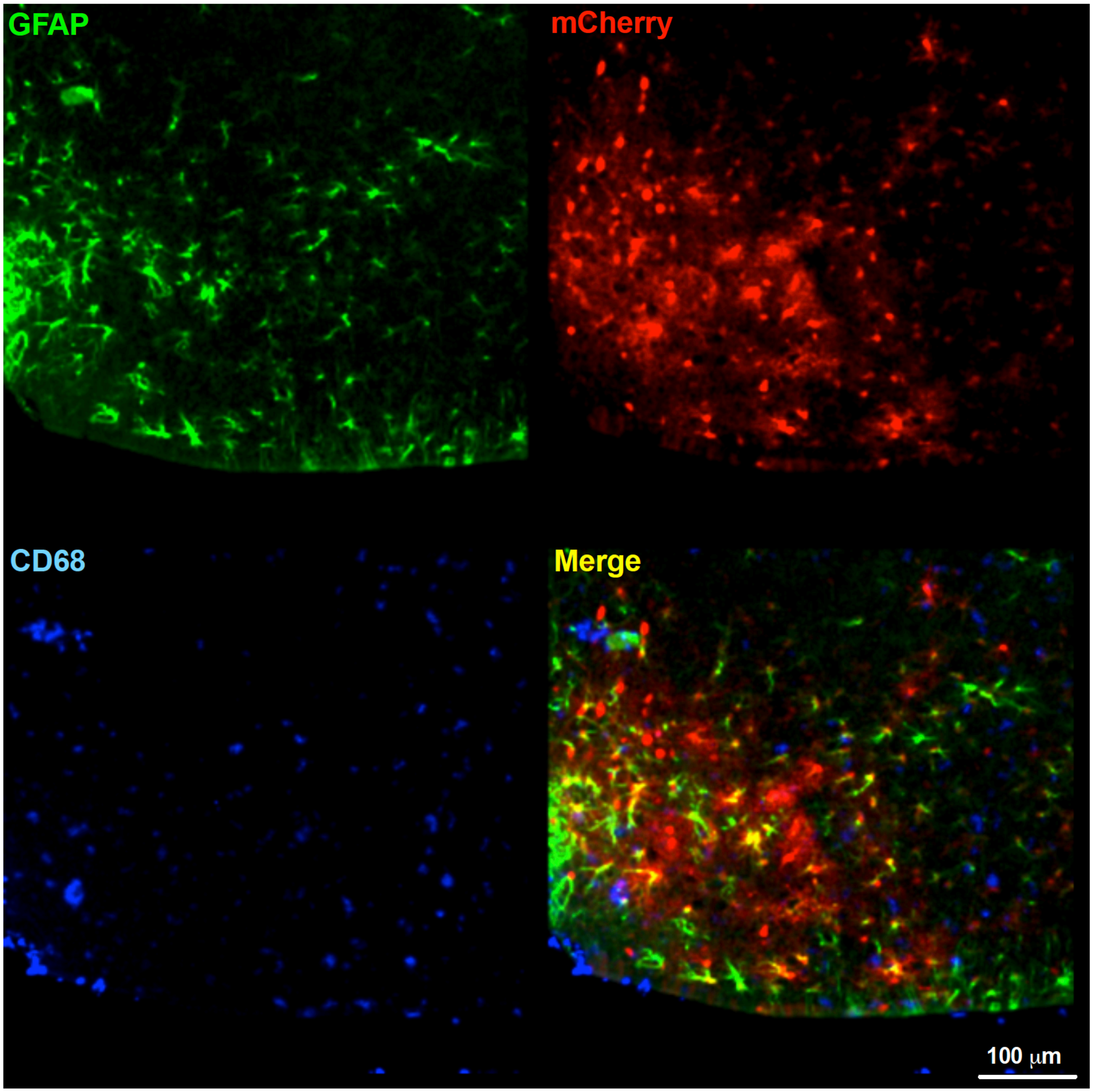

Supplementary Fig. 3 Photometry evidence of chemogenetic stimulation of ARH astrocytes. **a** Schematic illustration of viral transductions of astrocytes with GfaABC$_1$D-dependent hM3Dq and GCaMP$_{6f}$ and of in vivo calcium monitoring using fiber photometry. **b** A representative image of viral transfections in the ARH. **c** Representative images showing colocalizations of hM3Dq-mCherry (red) and GCaMP$_{6f}$ (green) in a selected region in the ARH. **d** Average GCaMP$_{6f}$ signaling recorded in the astrocyte hM3Dq-GCaMP$_{6f}$ co-transduced mice (n=6) receiving a single J60 i.p. injection. **e** Average GCaMP$_{6f}$ signaling recorded in the astrocyte hM3Dq-GCaMP$_{6f}$ co-transduced mice (n=4) receiving a single vehicle i.p. injection. **f** Average GCaMP$_{6f}$ signaling recorded in the astrocyte tdTomato-GCaMP$_{6f}$ co-transduced mice (n=6) receiving a single J60 i.p. injection. **g** Average GCaMP$_{6f}$ signaling recorded in the mice separately transduced with hM3Dq and GCaMP$_{6f}$ in the ARH astrocyte at the intervals of 10 days which received a single J60 i.p. injection (n=7). Scale bars for **b** 200 µm and for **c** 50 µm. Black arrows indicate i.p. injections of J60.

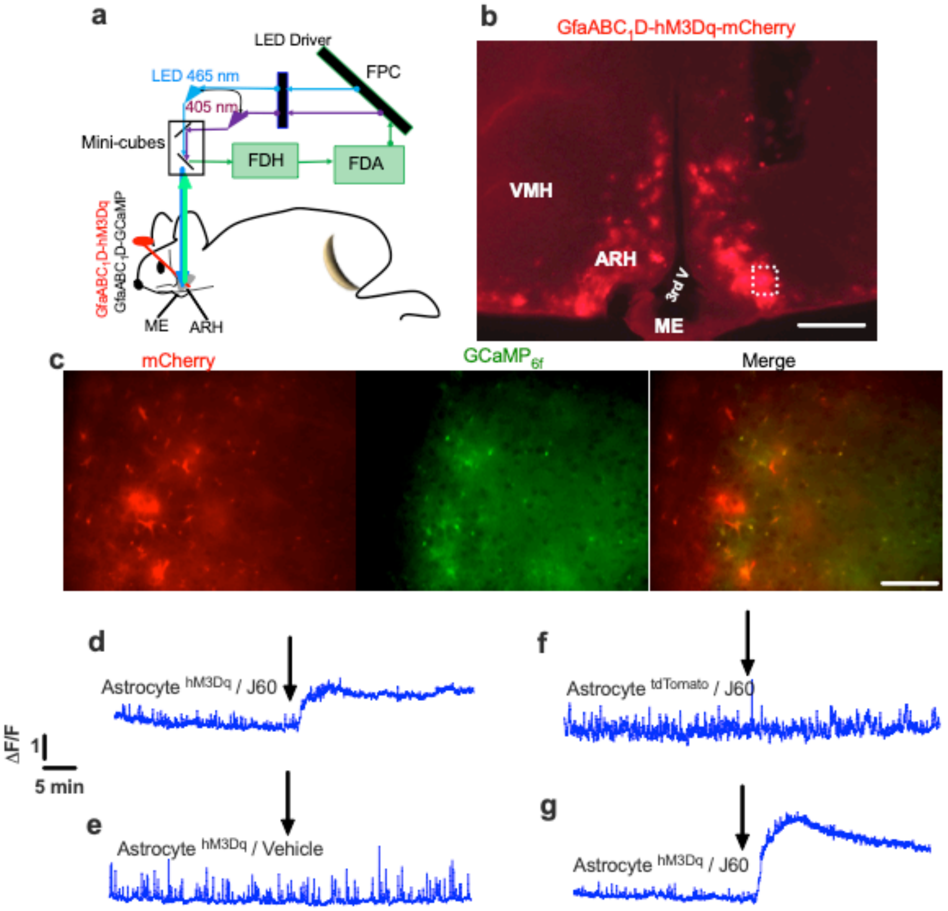

Supplementary Fig. 4 Both cold challenge and astrocyte stimulation elevate iWAT NE contents. a Group data of average intensity of iWAT GRAB_NE signals at ambient and cold (4 ^{o}C) temperature with vehicle (n=5) or αMPT (n=7) treatment, related to the text Figure 1b. **b**, **c** Group data of average intensity of iWAT GRAB_NE signals in the astrocyte **b** hM3Dq-or **c** tdTomato transduced mice (n=8 per group) compared to baseline, respectively related to the text Figure 2c and 2d. Two-way ANOVA with Sidak post hoc tests for **a**; two-tailed Student *t*-tests for **b** and **c** (p = 0.11); data represent mean ± s.e.m.; n.s. (Not significant).

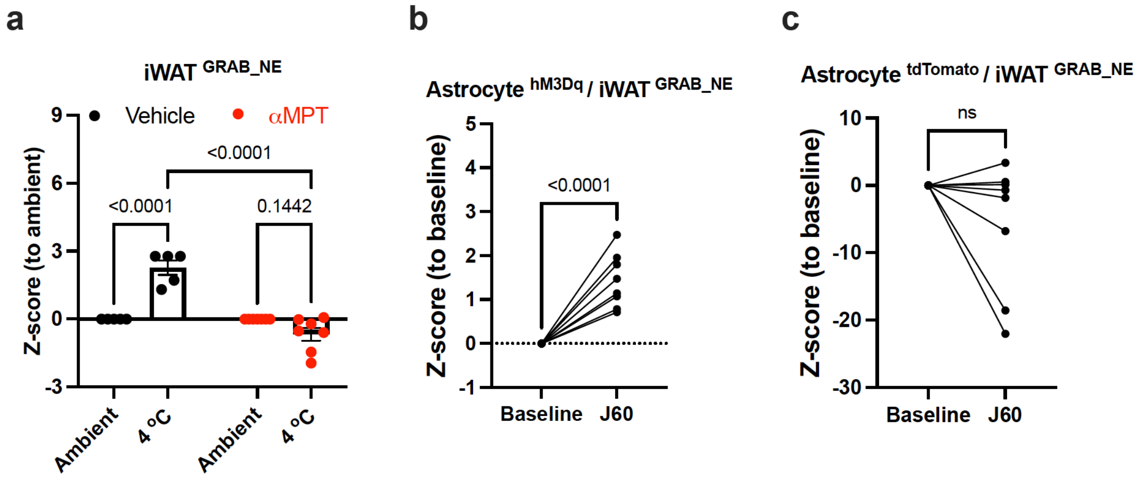

Supplementary Fig. 5 GFAP104-dependent vectors did not transduce microglia and neurons. Correlated to the text Figure 3; **a** Representative image of transfections of GFAP104-ChR2-mCherry vectors in the ARH; **b** Representative images of anti-CD68-labeled microglial cells (blue) and Alexa Fluor 488-conjugated anti-GFAP antibody labeled astrocytes (green) and virally ChR2-mCherry transduced cells (red). Almost all the mCherry-labelled cells were colocalized with GFAP-labeled cells but not those CD68 cells. **c** Representative images of virally mCherry transduced cells (red) and Alexa Fluor 488-conjugated anti-NSE antibody labeled neurons (green) and virally ChR2-mCherry transduced cells (red). No colocalizations of A488-labeld NSE neurons and mCherry-transduced cells were observed. Scale bars for **a** 500 µm, for **b** and **c** 10 µm.

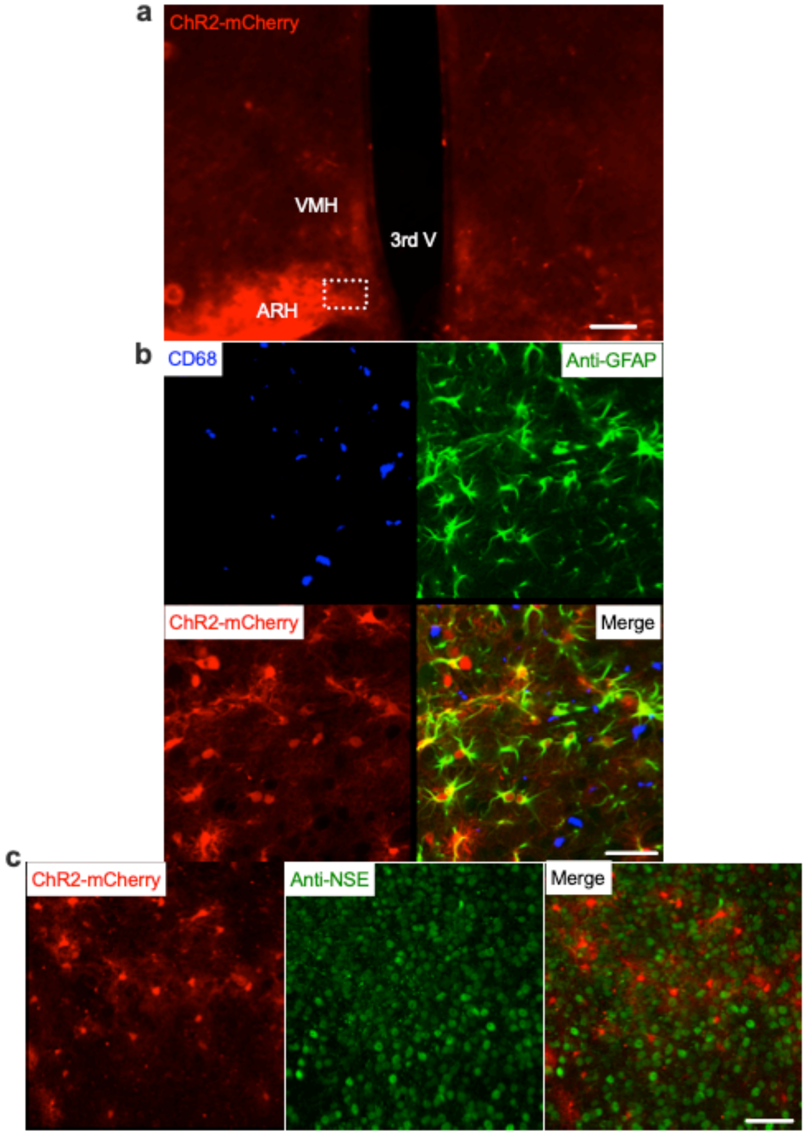

Supplementary Fig. 6 Morphological evidence of ChR2-mCherry expressions in T_{13} PG. Correlated to the text Figure 4e-i; **a** Representative image of $GCaMP_{6f}$ -positive neurons (green) and Cre-dependent expressions of ChR2-mCherry (red) in the virally injected T_{13} PG in dual TH-Cre $GCaMP_{6f}$ mice; **b** Representative images of $GCaMP_{6f}$ and mCherry expressions in iWAT in the same mice. Scale bars for **a** and **b** 50 μm.

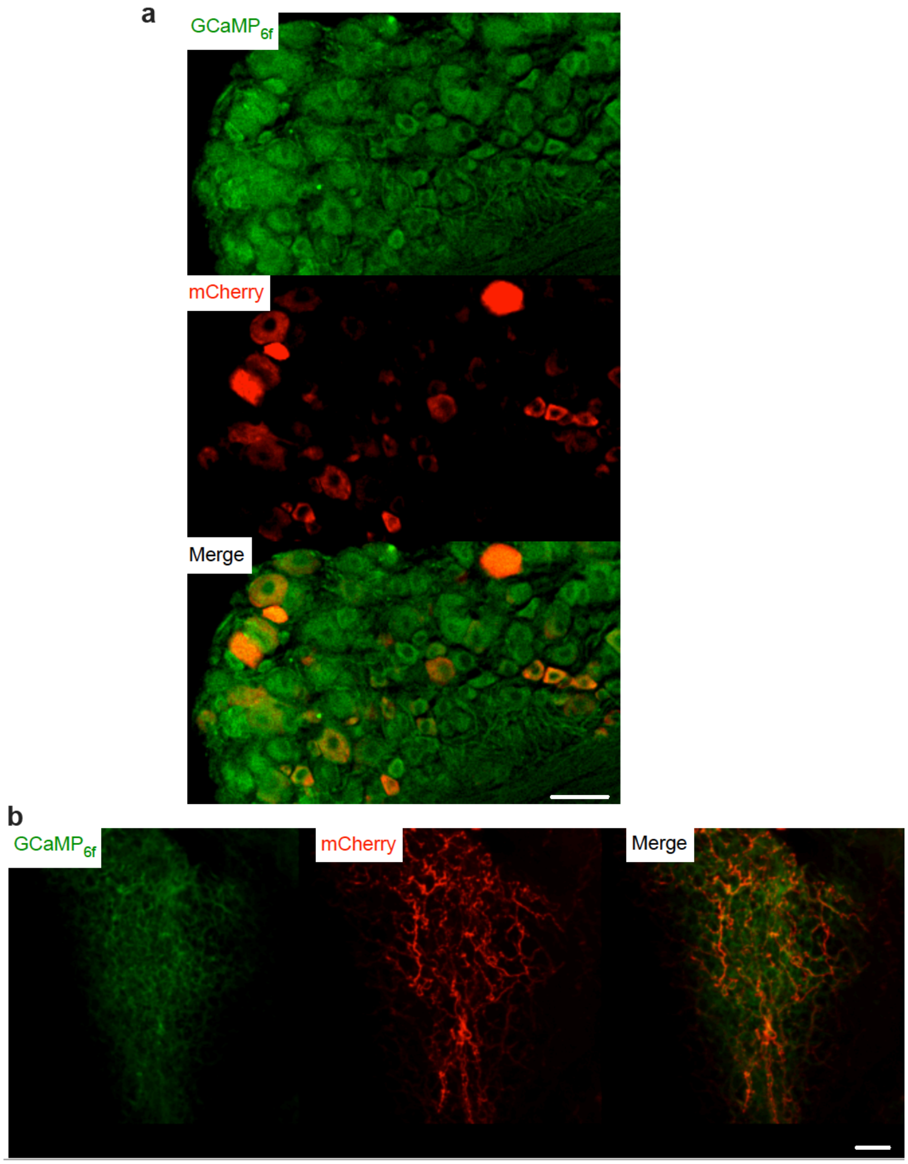

Supplementary Fig. 7 Astrocyte stimulation did not affect lipolytic and thermogenic gene expressions. a Group data of iWAT mRNA expressions of HSL and ATGL in the ARH astrocyte hM3Dq or tdTomato-transduced mice (n=6 per group), which received a single i.p. injection of J60 2 h before extracting the iWAT. **b** Group data of iWAT browning and thermogenic markers from the same mice. Two-tailed student *t*-tests; data represent mean ± s.e.m.; n.s. (Not significant).

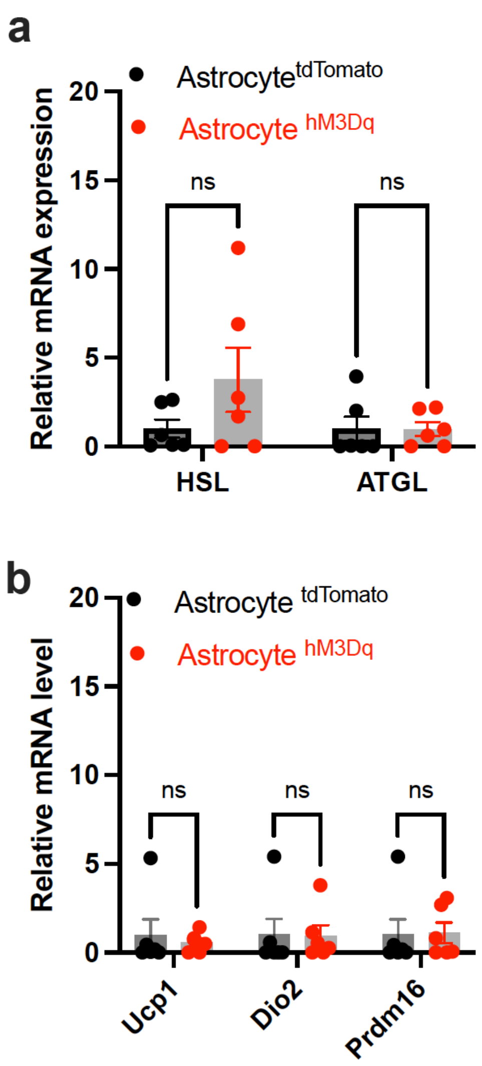

Supplementary Fig. 8 POMC neuron inhibition blunts astrocyte increasing the firing rates of local sympathetic inputs to iWAT. Group data of the electrical signals recorded on sympathetic inputs to iWAT in ARH astrocyte hM3Dq POMC neuron mCherry (n=7), astrocyte tdTomato POMC neuron hM4Di (n=8), or astrocyte hM3Dq POMC neuron hM4Di (n=7)-transduced POMC-Cre mice. Astrocytes were virally transduced with $GfaABC_1D$-dependent vectors and POMC neurons were transduced with Cre-dependent vectors in POMC-Cre mice. Mice were kept in a lightly sedated state. The nerves were placed on the recording electrodes as stated in the methods. Two-way ANOVA with Sidak post hoc tests; data represent mean ± s.e.m.; ** $p = 0.0017 < 0.01$; *** $p = 0.0008 < 0.001$; **** $p < 0.0001$.

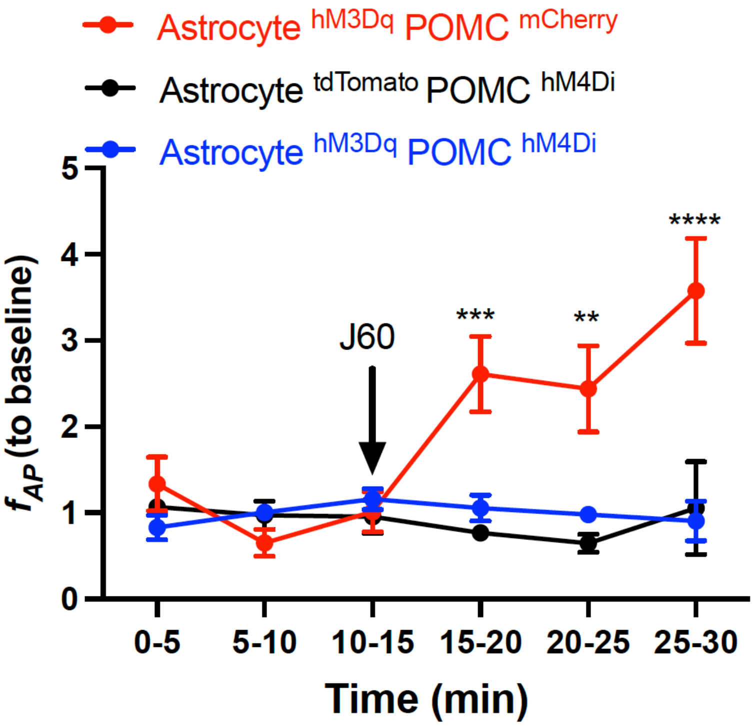

Supplementary Fig. 9 Chemogenetic inhibition of POMC neurons blunts the astrocyte stimulation-induced phosphorylation of HSL.

a Western blots of p-HSL (Ser660), HSL and actin in extracted iWATs from the ARH astrocyte tdTomato POMC neuron mCherry, astrocyte hM3Dq POMC neuron mCherry, or astrocyte hM3Dq POMC neuron hM4Di-transduced mice. Mice were treated with J60 via i.p. two h before extracting the iWAT for western blot assays. **b** Group data of p-HSL over HSL (n=3 per group). Astrocytes were virally transduced with $GfaABC_1D$-dependent vectors and POMC neurons were transduced with Cre-dependent vectors in POMC-Cre mice. One-way ANOVA; data represent mean ± s.e.m.; * $p = 0.02 < 0.05$.

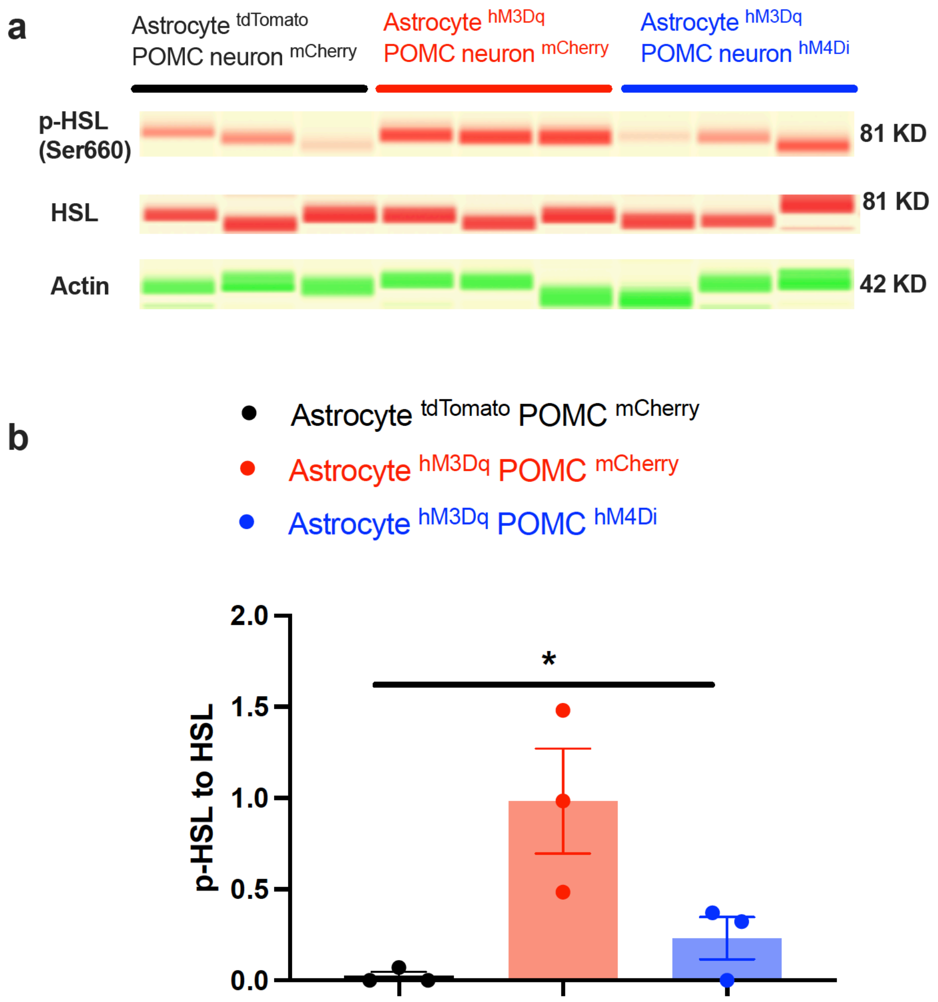

Supplementary Fig. 10 Viral transduction efficiency.

Group data of the total number of virally transduced ARH astrocytes of each animal in the representative hM3Dq (n=5) or ChR2 (n=4) transduced mice. Data represent mean ± s.e.m.

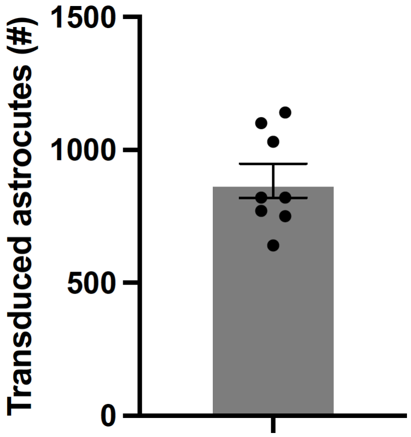

Source data of Supplementary Fig. 9a.

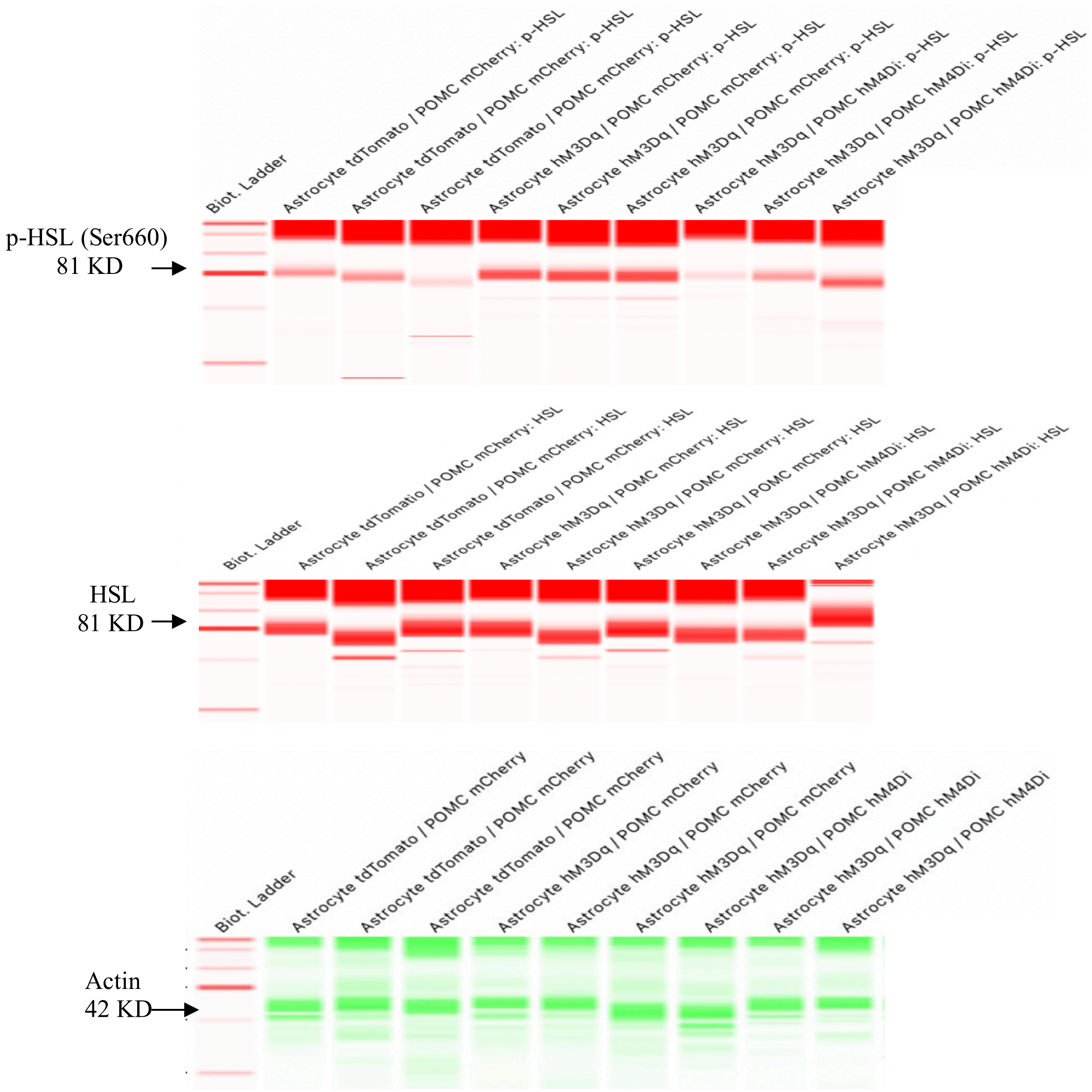

Supplement: Supplementary file 1 — Supplementary Information [file 41467_2022_35258_MOESM1_ESM.pdf]
