# Supplementary material for: Deconstruction of a hypothalamic astrocyte-white adipocyte sympathetic axis that regulates lipolysis in mice
Source: Nat Commun. 2022 Dec 7;13:7536. doi: 10.1038/s41467-022-35258-6 (PMC9729228; doi:10.1038/s41467-022-35258-6)
Supplement: Supplementary file 2 — Reporting Summary [file 41467_2022_35258_MOESM2_ESM.pdf]

nature portfolio

Corresponding author(s): Yang, Yunlei

Last updated by author(s): Nov 5, 2022

Reporting Summary

Nature Portfolio wishes to improve the reproducibility of the work that we publish. This form provides structure for consistency and transparency in reporting. For further information on Nature Portfolio policies, see our Editorial Policies and the Editorial Policy Checklist.

Statistics

For all statistical analyses, confirm that the following items are present in the figure legend, table legend, main text, or Methods section.

n/a	Confirmed	
☐	☒	The exact sample size (*n*) for each experimental group/condition, given as a discrete number and unit of measurement
☐	☒	A statement on whether measurements were taken from distinct samples or whether the same sample was measured repeatedly
☐	☒	The statistical test(s) used AND whether they are one- or two-sided<br>*Only common tests should be described solely by name; describe more complex techniques in the Methods section.*
☒	☐	A description of all covariates tested
☐	☒	A description of any assumptions or corrections, such as tests of normality and adjustment for multiple comparisons
☐	☒	A full description of the statistical parameters including central tendency (e.g. means) or other basic estimates (e.g. regression coefficient) AND variation (e.g. standard deviation) or associated estimates of uncertainty (e.g. confidence intervals)
☐	☒	For null hypothesis testing, the test statistic (e.g. F, t, r) with confidence intervals, effect sizes, degrees of freedom and P value noted<br>*Give P values as exact values whenever suitable.*
☒	☐	For Bayesian analysis, information on the choice of priors and Markov chain Monte Carlo settings
☒	☐	For hierarchical and complex designs, identification of the appropriate level for tests and full reporting of outcomes
☒	☐	Estimates of effect sizes (e.g. Cohen's *d*, Pearson's *r*), indicating how they were calculated

Our web collection on statistics for biologists contains articles on many of the points above.

Software and code

Policy information about availability of computer code

Data collection	GCaMP6f and GRAB_NE signals were collected using a tethered two-channel dual-wavelength fiber photometry (FP) system (Doric Lenses) or a wireless TeleFipho (Amuza); Histological images were taken using a confocal microscopy (Leica SP8) and/or an inverted microscopy (Olympus IX51); Electrophysiological recordings of iWAT sympathetic nerves were performed using an electrophysiology rig (Molecular Devices, MultiClamp 700B); Photostimulation was performed using blue lasers (CrystLaser 473 nm); Adenosine in the ARC was evaluated using ADO biosensors and potentiostate (Model 8102; Pinnacle Tech); NE in the iWAT was measured using the carbon fiber electrodes (7004-CFE) and the FSCV system (Pinnacle Tech); Transgenic mice were genotyped using the Gel Documentation Systems (Corning). All other information such as western blots, RT-qPCR, IF, and mouse surgeries is detailed in the Methods.
Data analysis	Fiber photometry data were analyzed using a Doric Neuroscience Studio Software (V5.3.3.14). Electrophysiology data were analyzed using Molecular Devices pClampfit software (V11.0) as stated in the Method section. ADO and NE data were analyzed using the Sirenia Acquisition (V2.2.5) and FSCV software (2.0.9)(Pinnale Technology) respectively. Histological images were analyzed using the Leica Application Suite X. Prism 9.0 (GraphPad Software) was used to for statistical analysis. Student's t-tests were used to analyze differences between two groups of the same or different mice when appropriate, respectively. One-way ANOVA with post hoc test was used to compare group data from more than two groups of mice. Two-way ANOVA with the within-subject factors of time segment and treatment or mixed ANOVA with the within-subject factor of time segment and the between-subjects factor of viral injections type were used to analyze data from more than two groups across various time points.

For manuscripts utilizing custom algorithms or software that are central to the research but not yet described in published literature, software must be made available to editors and reviewers. We strongly encourage code deposition in a community repository (e.g. GitHub). See the Nature Portfolio guidelines for submitting code & software for further information.

Data

Policy information about availability of data

All manuscripts must include a data availability statement. This statement should provide the following information, where applicable:

- Accession codes, unique identifiers, or web links for publicly available datasets
- A description of any restrictions on data availability
- For clinical datasets or third party data, please ensure that the statement adheres to our policy

All the data generated and analyzed that support the findings in this study are within the article and its supplementary information files, and are available from the corresponding author upon reasonable request. Source data are provided with this paper.

Human research participants

Policy information about studies involving human research participants and Sex and Gender in Research.

Reporting on sex and gender	N/A
Population characteristics	N/A
Recruitment	N/A
Ethics oversight	N/A

Note that full information on the approval of the study protocol must also be provided in the manuscript.

Field-specific reporting

Please select the one below that is the best fit for your research. If you are not sure, read the appropriate sections before making your selection.

☒ Life sciences ☐ Behavioural & social sciences ☐ Ecological, evolutionary & environmental sciences

For a reference copy of the document with all sections, see nature.com/documents/nr-reporting-summary-flat.pdf

Life sciences study design

All studies must disclose on these points even when the disclosure is negative.

Sample size	The sample sizes used in this study were chosen on standard sizes in the fields and based on our previous studies (such as Yang L et al., Cell Reports, 2015, PMID#25921535; Zhang J et al., Nat Commun 2020, PMID#33303759; Zhang J et al., Mol Psychiatry, 2021, PMID#33067580.
Data exclusions	Following post hoc histological conformation of viral transfection and cannula placements, all mice with inaccurate viral infections or cannula placements were excluded from the experiments. Only mice with accurate viral injections and cannula placements were included in data analysis.
Replication	All experiments were successfully repeated at least twice for each mouse and average values were calculated for each individual mouse for statistical analysis.
Randomization	Age and sex-matched were randomly assigned to experimental or control groups.
Blinding	The experimenters were not blind to transgenic animals (TH-Cre; POMC-Cre; GCaMP6f) and the identification of viral vectors because the experimenters need to label the transgenic mice and injected vectors. However, the experimenters were blind to the identification of pharmacological reagents (i.e. J60 vs vehicle) when they performed the experiments. The data were also analyzed blind.

Reporting for specific materials, systems and methods

We require information from authors about some types of materials, experimental systems and methods used in many studies. Here, indicate whether each material, system or method listed is relevant to your study. If you are not sure if a list item applies to your research, read the appropriate section before selecting a response.

Materials & experimental systems

n/a	Involved in the study
☐	☒ Antibodies
☒	☐ Eukaryotic cell lines
☒	☐ Palaeontology and archaeology
☐	☒ Animals and other organisms
☒	☐ Clinical data
☒	☐ Dual use research of concern

Methods

n/a	Involved in the study
☒	☐ ChIP-seq
☒	☐ Flow cytometry
☒	☐ MRI-based neuroimaging

Antibodies

Antibodies used

Primary rabbit Alexa fluor 488-conjugated anti-GFAP antibodies (bs-0199R-A488; Bioss);
Primary mouse Alexa fluor 488-conjugated anti-NSE antibodies (bsm-33072M-A488; Bioss);
Primary rabbit Alexa fluor 350-conjugated anti-CD68 antibodies (bs-0649R-A350; Bioss);
Primary rabbit anti-pHSL (Ser660) antibodies (PA5-64494; Invitrogen);
Primary mouse Alexa fluor 647-conjugated anti-TH antibodies (sc-25269-AF647; Santa Cruz);
Primary rabbit Alexa fluor 350-conjugated anti-TH antibodies (bs-0016R-350; Bioss);
Primary rabbit anti-HSL antibodies (PA5-17196; Invitrogen);
Secondary IRDye 800 CWdonkey anti-rabbit IgG (#926-32213; Li-Cor);
Secondary anti-rabbit NIR antibody (#043-819; ProteinSimple);
Secondary anti-rabbit IR antibody (#043-822; ProteinSimple).

Validation

The antibodies listed in the above have been in common application and have been validated in literature for use in mouse. The detailed information have been provided in the company's websites. We also confirmed that the antibodies stained in an expected pattern and distributed in the target proteins.
Primary rabbit Alexa fluor 488-conjugated anti-GFAP antibodies (bs-0199R-A488; Bioss);
(https://www.biossusa.com/products/bs-0199r-a488; Ref: Sweeney P et al (2016) GLIA, PMID#27658520)
Primary mouse Alexa fluor 488-conjugated anti-NSE antibodies (bsm-33072M-A488; Bioss);
(https://www.biossusa.com/products/bsm-33072m-a488)
Primary rabbit Alexa fluor 350-conjugated anti-CD68 antibodies (bs-0649R-A350; Bioss);
(https://www.biossusa.com/products/bs-0649r-a350)
Primary rabbit anti-pHSL (Ser660) antibodies (PA5-64494; Invitrogen);
(https://www.thermofisher.com/antibody/product/Phospho-HSL-Ser660-Antibody-Polyclonal/PA5-64494)
Primary mouse Alexa fluor 647-conjugated anti-TH antibodies (sc-25269-AF647; Santa Cruz);
(https://www.scbt.com/p/th-antibody-f-11?requestFrom=search)
Primary rabbit Alexa fluor 350-conjugated anti-TH antibodies (bs-0016R-A350; Bioss);
(https://www.biossusa.com/products/bs-0016r-a350)
Primary rabbit anti-HSL antibodies (PA5-17196; Invitrogen);
(https://www.thermofisher.com/antibody/product/HSL-Antibody-Polyclonal/PA5-17196).

Animals and other research organisms

Policy information about studies involving animals; ARRIVE guidelines recommended for reporting animal research, and Sex and Gender in Research

Laboratory animals

Male and female (age 8-12 weeks) wild-type C57BL/6J, POMC-Cre, TH-Cre, and Ai95D mice have been described previously and purchased from The Jackson Laboratory. Mice were group-housed 3-5 mice per cage in ambient temperature (22-25 oC)- and humidity-controlled rooms on a 12-h light:12-h dark cycle, with lights on from 8:00 a.m. to 8:00 p.m., and with ad libitum access to water and mouse regular chow. Mice were single-caged after they received viral transductions with or without guide cannula insertion until all experimental procedures were finished. The virally transduced mice were randomly assigned to experimental and control groups at the start of experiments. Mice were also randomly assigned and evenly age and sex-matched for the different viral injections and treatments as described in the text and figure legends.

Wild animals

No wild animals were used in this study.

Reporting on sex

As stated above, sex-matched (half and half for each group unless where noted in the text and figure legends) were randomly assigned to experimental and control groups.

Field-collected samples

No field-collected samples were used in this study.

Ethics oversight

Experimental protocols were conducted according to U.S. National Institutes of Health guidelines for animal research, and were approved by our Institutional Animal Care and Use Committees.

Note that full information on the approval of the study protocol must also be provided in the manuscript.
